# Social connectedness as a determinant of mental health: A scoping review

**DOI:** 10.1371/journal.pone.0275004

**Published:** 2022-10-13

**Authors:** Priya J. Wickramaratne, Tenzin Yangchen, Lauren Lepow, Braja G. Patra, Benjamin Glicksburg, Ardesheer Talati, Prakash Adekkanattu, Euijung Ryu, Joanna M. Biernacka, Alexander Charney, J. John Mann, Jyotishman Pathak, Mark Olfson, Myrna M. Weissman

**Affiliations:** 1 Department of Psychiatry, Vagelos College of Physicians and Surgeons, Columbia University Irving Medical Center, New York, NY, United States of America; 2 Division of Translational Epidemiology, New York State Psychiatric Institute, New York, NY, United States of America; 3 Departments of Psychiatry and Genetics & Genomic Sciences, Icahn School of Medicine at Mount Sinai, New York, NY, United States of America; 4 Division of Health Informatics, Department of Population Health Sciences, Weill Cornell Medicine, New York, NY, United States of America; 5 Department of Information Technologies and Services, Weill Cornell Medicine, New York, NY, United States of America; 6 Department of Health Sciences Research, Mayo Clinic, Rochester, MN, United States of America; 7 Division of Molecular Imaging and the Neuropathology, Departments of Psychiatry and Radiology, New York State Psychiatric Institute, Vagelos College of Physicians and Surgeons, Columbia University Irving Medical Center, New York, NY, United States of America; Texas State University, UNITED STATES

## Abstract

Public health and epidemiologic research have established that social connectedness promotes overall health. Yet there have been no recent reviews of findings from research examining social connectedness as a determinant of mental health. The goal of this review was to evaluate recent longitudinal research probing the effects of social connectedness on depression and anxiety symptoms and diagnoses in the general population. A scoping review was performed of PubMed and PsychInfo databases from January 2015 to December 2021 following PRISMA-ScR guidelines using a defined search strategy. The search yielded 66 unique studies. In research with other than pregnant women, 83% (19 of 23) studies reported that social support benefited symptoms of depression with the remaining 17% (5 of 23) reporting minimal or no evidence that lower levels of social support predict depression at follow-up. In research with pregnant women, 83% (24 of 29 studies) found that low social support increased postpartum depressive symptoms. Among 8 of 9 studies that focused on loneliness, feeling lonely at baseline was related to adverse outcomes at follow-up including higher risks of major depressive disorder, depressive symptom severity, generalized anxiety disorder, and lower levels of physical activity. In 5 of 8 reports, smaller social network size predicted depressive symptoms or disorder at follow-up. In summary, most recent relevant longitudinal studies have demonstrated that social connectedness protects adults in the general population from depressive symptoms and disorders. The results, which were largely consistent across settings, exposure measures, and populations, support efforts to improve clinical detection of high-risk patients, including adults with low social support and elevated loneliness.

## Introduction

While there is no universally accepted definition of social connectedness, it generally denotes a combination of interrelated constructs spanning social support, social networks, and absence of perceived social isolation [1]. There is a broad-based agreement in the public health and epidemiologic literature that social connectedness protects and promotes mental and physical health and decreases all-cause mortality [1–3]. Researchers in fields ranging from psychology and epidemiology to sociology have been aware of these findings for several decades, but its implications have only recently begun to be appreciated more widely [4]. A Mendelian randomization study has validated the protective effects of trusted social connections on depression [5]. In a recent study of 100,000 participants in the UK Biobank [6], frequency of confiding in others and visits with family and friends emerged from over 100 modifiable risk factors as the strongest predictor of depression. This suggests that social connectedness may have protective effects or may be modified by development of a mood disorder. There is now a social connection domain in the Epic Social Determinants of Health wheel included in Electronic Health Records used in many major health organizations.

In light of these developments, we decided to conduct a scoping review of the relevant literature published between 2015 and 2020 to evaluate the extent to which social connectedness influences risk for depression and anxiety. We also sought to determine which aspects of social connectedness, namely social networks, social support) are most protective. There have been a myriad of theories explaining the association between social connectedness and mental health. These include, but not limited to Bowlby attachment theory [7,8], social support and buffering theory [9], stress-buffering theory [10], and social support resource theory [11]. According to attachment theory, depression and despair develop because of a break in attachments to people you feel close to, in the context of deaths, disputes, life changes, loneliness, or the absence of attachments [7,8]. Another relevant theory that accounts for this association is Cohen et al’s exposition of social support and buffering theory [9], an extension of Lazarus’ general stress and coping theory [12], which underscored the beneficial effects of received and perceived social support against the negative impact of stressful events without specifying the types of relationships involved. These theoretical underpinnings provided an important framework for this review that synthesizes recent literature on the various categories of social connectedness and their differential effects on depression and anxiety in specific populations. Because depression and anxiety can have adverse effects on social connectedness [13], we restricted our search to longitudinal/cohort studies from which appropriate temporal ordering that is necessary, although not sufficient, for causal inferences, can be established. We performed a scoping review of the literature published during the last five years addressing whether social connectedness is longitudinally associated with common mental health outcomes of depression and anxiety among adults.

## Methods

**Fig 1:**
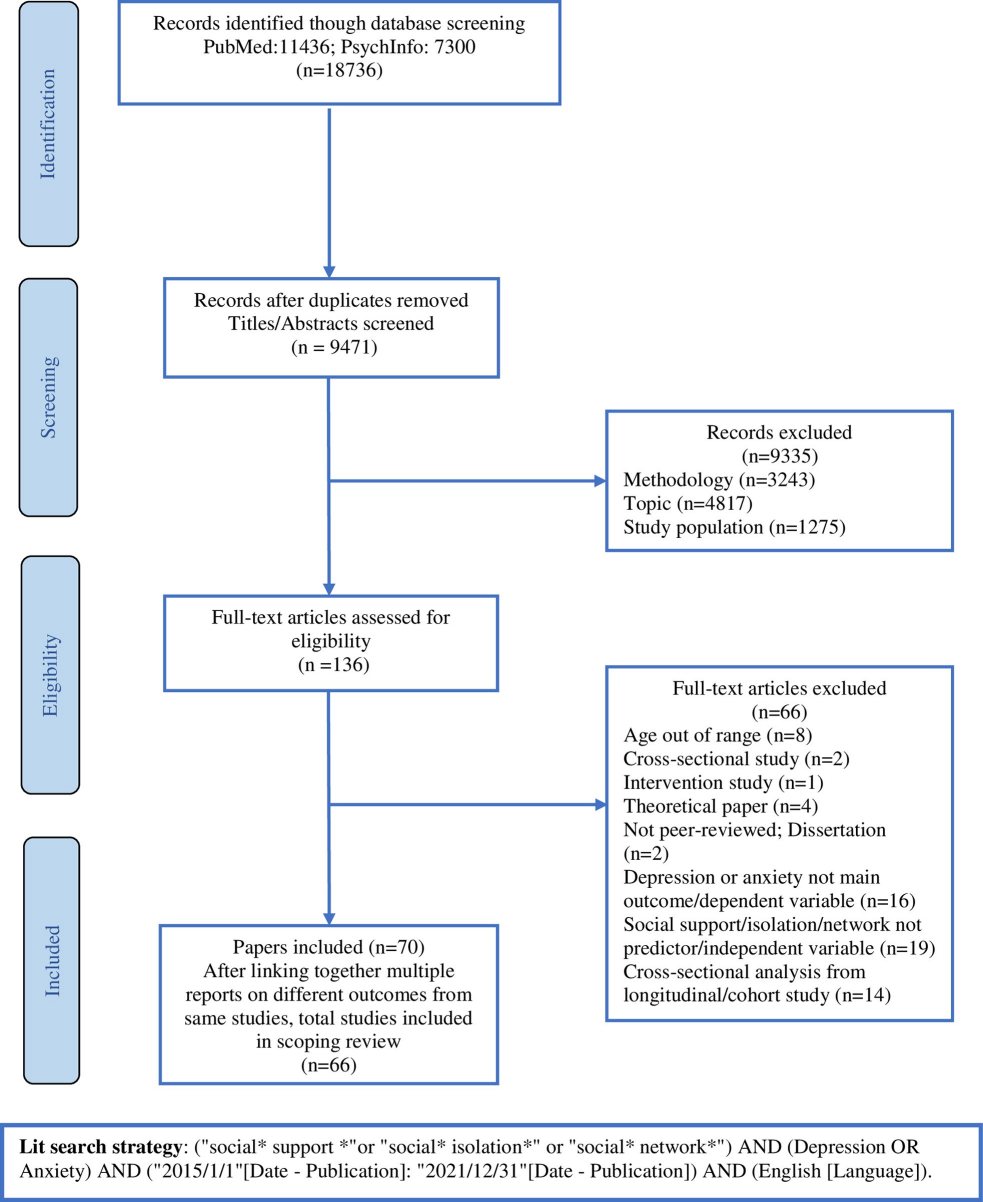
PRISMA flow chart of the scoping review. PRISMA diagram showing search and selection process of scoping review.

Searches of PubMed and PsychInfo databases and inspection of reference lists of relevant papers published during January 2015 and December 2021 were conducted following the Preferred Reporting Items for Systematic Reviews and Meta-Analyses Extension for Scoping Review (PRISMA-ScR) guidelines. The databases were searched using the following search strategy: ("social* support *"or "social* isolation*" or "social* network*") AND (Depression OR Anxiety) AND ("2015/1/1"[Date—Publication]: "2021/12/31"[Date—Publication]) AND (English [Language]). Fig 1 presents a flow diagram displaying the process of searching and selecting the studies.

### Search strategy and selection criteria

For inclusion in the review, studies were required to meet the following criteria: (a) employed longitudinal/cohort study design; (b) published in peer-reviewed journals between the years 2015 and 2021 in English; (c) assessed social support, social networks, or social isolation as one of the main predictor variables; (d) the mental health outcomes analyzed in the articles had to be either depression or anxiety; and (e) recruited participants aged 18 years or older in the study.

Studies were excluded if: (a) the article did not report original data (e.g., the article was a theoretical paper, review paper, or meta-analysis); (b) social connectedness as operationalized in the review was not measured as a predictor variable; (c) the sample included participants with pre-existing health conditions (e.g., HIV, chronic disease, cancer, stroke, etc.) except mental health conditions that are generally comorbid with depression or anxiety; and (d) the study did not focus on adults.

### Data extraction

After removing irrelevant titles and duplicates, the remaining articles were reviewed with respect to the eligibility criteria. Two authors (TY and PW) scrutinized titles and abstracts, and full text articles potentially eligible for this review were obtained. Key information from included papers was initially extracted and tabulated by one author (TY). The accuracy of this information was independently verified by another co-author (PW). Discrepancies were resolved through consensus. The data extracted included basic descriptive information about the sample, study type, length of follow-up, relevant predictor and outcome measures, and main findings. In cases where the same parent study data were employed in more than one publication, the papers were considered one study. Due to the wide variation in study designs and populations, we did not attempt meta-analysis, but rather provide a narrative synthesis of the main findings.

## Results

The initial search yielded 18,736 articles, and 9471 articles were retained after duplicates and irrelevant articles were removed. After title and abstract screening, 136 articles were assessed for eligibility. Among studies selected for full-text review, 6 articles were deemed ineligible and excluded for various reasons: 17 did not employ longitudinal study design, 8 recruited participants aged younger than 18 years, 4 were theoretical papers, 19 did not assess social support, social networks, and/or social isolation as predictor variable, 16 did not have depression or anxiety as the main outcome variable, and 2 were not from peer-reviewed sources. The PRISMA flowchart (Fig 1) provides further detail on reasons for exclusion. Articles that used data from the same study have been combined in the table and were considered as one study. A total of 70 articles representing 66 unique studies that met inclusion criteria were included in the review.

### Study characteristics

Data from 66 unique articles were categorized into three tables. Tables 1, 2 and 3 present data separately for social support, social isolation/loneliness, and social networks. Table 1 was further divided into two sections: Table 1A includes studies that investigated longitudinal effects of social support on depression or anxiety in samples other than pregnant women, and Table 1B includes studies with pregnant women.

**Table 1:** a. Characteristics of included studies on social support for nonpregnant samples. b. Characteristics of included studies on social support for pregnant women.

Reference	Sample/Setting [Country] Age (y)	Study Type	Follow-up Times	Social Support [Measure]	MH Outcome [Measure]	Main Findings for Social Support
Åhlin et al., 2018 [14]	n = 6679 workers [Sweden] Age = 16–64	Data from Swedish Longitudinal Occupational Survey of Health (SLOSH)	6 waves 2006, 2008, 2010, 2012, 2014, 2016	Workplace social support [DCSQ]	Depressive symptoms [SCL-CD6]	Perceiving low social support is associated with subsequent higher or increasing levels of depressive symptoms over time.
Ahmad et al., 2021 [15]	n = 1924 refugees [Canada] Mean Age: 38.5	Data from Syrian Refugee Integration and Long-term Health Outcomes in Canada study (SyRIA.lth)	2 timepoints T1: baseline T2: 1-year later	Perceived social support [MSPSS]	Depressive symptoms [PHQ-9]	One of the factors significantly associated with moderate- and severe-level of depression symptoms at year 2 was lower perceived social support.
Aroian et al., 2017 [16]	n = 388 married Arab immigrant women [USA] Mean Age: 42	Longitudinal study	3 waves roughly 18 months apart	Perceived social support [MSPSS]	Depressive symptoms [CES-D]	An increased rate of change over time in friend support contributed to lower depression at Time 3, but changes over time in support from husband and support from family were not significant predictors of depression at Time 3.
Berthelsen et al., 2015 [17]	n = 2059 nurses [Norway] Age: 21–39	One-year follow-up study	2 timepoints T1: baseline T2: 1-year follow-up	Social support [DCSQ]	Anxiety and depression symptoms [HADS]	Structural equation modeling revealed statistically significant reverse regression paths between baseline symptoms of anxiety and depression and follow-up role clarity, role conflict, fair leadership, and social support.
Billedo et al., 2019 [18]	n = 98 international students from 76 host countries Age: 16–49	Longitudinal study	3 waves with 3-month intervals between each wave	Perceived social support [SPS]	Depressive symptoms [CES-D-11]	Face to face interaction with the host-country network had immediate positive impacts on international students perceived social support, which in turn, predicted lower depressive symptoms
Boyden et al., 2020 [19]	n = 200 parents of 158 seriously ill children [USA] Age: ≥18	Prospective cohort study: Decision Making in Serious Pediatric Illness study	3 timepoints T1: baseline T2: 12 months T3: 24 months	Perceived social support [SPS]	Prenatal anxiety [HADS]	Cross-sectionally, social support scores were negatively associated with anxiety scores at each time point. Longitudinally, social support scores were associated with anxiety scores, although this association weakened in adjusted modeling.
Canavan et al., 2021 [20]	n = 1474 participants [USA] Age: 32–87	National longitudinal study: Americans’ Changing Lives (ACL) data set	4 waves 1986, 1989, 1994, 2002	Social support [3 standardized component indices that correspond to positive support from: spouse, child/children, friend/relative]	Depressive symptoms [CES-D]	Social support buffered the relationship between involuntary job loss and depressive symptoms among a subgroup of individuals who were more likely to be White, higher educated, and have higher social support before job loss.
Ciarleglio et al., 2018 [21]	n = 375 active-duty veterans deployed to Iraq at least once between 2003 and 2005 [USA] Age: ≥ 21	Longitudinal study: VA Cooperative Studies Program Study #566 (CSP#566)	3 timepoints T1: prior to deployment T2: during deployment T3: after return from deployment	Post-war-zone social support [DRRI]	Depression and anxiety severity [DASS-21 Generalized anxiety disorder [MINI]	Higher scores on the post-deployment social support scale were associated with lower risk of all outcomes except problem drinking. Post-deployment social support remained a strong protective factor for PTSD, depression, and anxiety symptom severity at long-term follow-up.
Crowe et al., 2016 [22]	n = 6521 participants in early twenties [Australia] Age: 20–24	Longitudinal study: Personality and Total Health (PATH) Through Life Project	3 waves with 4-year intervals between each wave over 8-year period	Level of positive social support [2 sets of 5-item questions]	Depressive symptoms [Goldberg Depression Scale]	Social support, financial hardship, and a sense of personal control (mastery) all emerged as important mediators between unemployment and depression.
Feldman et al., 2021 [23]	n = 135 emergency medical service providers [USA] Mean Age: 35.63	Longitudinal study	Baseline and 3-month follow-up	Perceived quality of relationships and support [Interpersonal Support Evaluation List]	PTSD [PCL-C] Depression [CES-D] Anxiety [BAI]	Lower social support and poor sleep hygiene at baseline predicted increases in depressive symptoms, PTSD symptoms, and anxiety symptoms at 3-month follow up.
Handley et al., 2019 [24]	n = 2639 rural residents [Australia] Age: 18–47	Data from Australian Rural Mental Health Study (ARMHS)	4 timepoints T1: baseline T2: 1 year T3: 3 years T4: 5 years after baseline	Perceived interpersonal support [Interview Schedule for Social Interaction]	Depression [PHQ-9]	The baseline-only model found that the odds of depression were increased for those who were permanently unable to work, had low perceived availability of interpersonal support, had a greater number of recent adverse life events, and had higher levels of neuroticism.
Haverfield et al., 2019 [25]	n = 406 patients with co-occurring mental health and SUDs [USA] Age: NR	Longitudinal study	4 timepoints T1: baseline T2: 3-, T3: 9-, and T4: 15-month follow-ups	Social support [Subscale of Basic Need Satisfaction Scale]	Depression severity [PHQ-9]	Less family support (i.e., more conflict) was the most consistent predictor of mental health and substance use outcomes and was associated with greater psychiatric, depression, PTSD, and drug use severity.
Hayslip et al., 2015 [26]	n = 86 grandparent caregivers [USA, Canada] Age: 38–90	Longitudinal study	2 timepoints over 1-year time frame	Perceived social support [MSPSS]	Depressive symptoms [CES-D]	The interaction of overall health and social support at Time 1 predicted Time 2 depression. For those who lacked social support, overall health was negatively related to depression symptoms 1 year later.
Houtjes et al., 2017 [27]	n = 277 older adults [Amsterdam] Age: >55	Data of the Longitudinal Aging Study Amsterdam (LASA)	16 timepoints/ observations covering 13 years	Social support [Self-reported questionnaire]	Depressive symptoms [CES-D]	A 2‐way interaction between depression course types and time showed significant differences in instrumental support received over time in older people with a late‐life depression.
Misawa et al., 2019 [28]	n = 3464 elderly people [Japan] Age: ≥ 65	Longitudinal panel data: Part of Aichi Gerontological Evaluation Study (AGES) project	2 waves. 2003, 2006–2007	Social support [Self-reported questionnaire]	Depression [GDS-15]	The frequency of meeting with friends and self-rated health predicted reduced odds of depression in men, while age predicted increased odds in women.
Noteboom et al., 2016 [29]	n = 1085 respondents from health care settings [Netherlands] Age: > 18	Longitudinal cohort study: Netherlands Study of Depression and Anxiety (NESDA)	2 timepoints T1: baseline T2: 2-year follow-up	Social support [CPQ]	Depressive disorders [CIDI]	Contrary to authors’ expectations, low perceived support, or the perceived aspects (perceived emotional support or negative aspects of support) are not associated with the development of a new episode of depression after accounting for baseline clinical characteristics
Van Den Brink et al., 2018 [30]	n = 1474 patients with MDD Sample 1: 1115 patients Sample 2: 359 patients [Netherlands] Age: 18–90	Data from two cohort studies Sample1: NESDA Sample 2: Netherlands Study of Depression in Older Persons (NESDO)		Social support received from partner and from closest friend or family member [CPQ]	Presence of depression [CIDI] Depression severity [IDS-SR]	Negative experiences with social support were the only social relational variable, which independently predicted non-remission of depression at follow-up.
Porter et al., 2017 [31]	n = 343 undergraduates and their romantic partners [USA] Age: 18–23	Longitudinal study	2 timepoints T1: baseline T2: 12-month follow-up	Perceived social support [MSPSS]	Social anxiety [SIAS] Depression, anxiety symptoms [DASS-21]	Social anxiety is not associated with less support as rated by observers. Socially anxious individuals received less support from their partners according to participant but not observer report.
Scardera et al., 2020 [32]	n = 1174 emerging adults [Canada] Age:19–20	Population-based cohort study: Data from Quebec Longitudinal Study of Child Development	2 timepoints T1: baseline T2: 1 year later	Perceived social support [SPS-10]	Depressive symptoms [CES-D] Anxiety symptoms [GAD-7]	Perceived social support was significantly associated with fewer depressive and anxiety symptoms, and suicide-related outcomes at 1-year follow-up. The magnitude of these associations appears stronger for depressive symptoms compared with anxiety symptoms.
Souto et al., 2021 [33]	n = 15105 civil servants [Brazil] Age:35–74	Multicenter cohort: Longitudinal Study of Adult Health (ELSA-Brasil)	2 waves 2008–2010 2012–2014	Social capital- resource available on social networks [Resource Generator]	Depressive episodes [CIS-R]	Low social capital in the “social support” dimension was associated with the incidence of depressive episodes (RR = 1.66; 95% CI: 1.01–2.72) among men. Social support was associated with the maintenance of depressive episodes (RR = 2.66; 95% CI: 1.61–4.41) among women.
Stafford et al., 2019 [34]	n = 7171 people aged 50 and older living in [England] Age: >50	Data from the English Longitudinal Study of Ageing (ELSA)	5 waves 2002, 2004, 2006, 2008, 2010	Partner/spouse support [3-items questionnaire]	Depressive symptoms [CES-D-8]	Greater increases over time in depressive symptoms were seen in those with lower positive support at baseline. More baseline depressive symptoms predicted greater declines in positive support and greater increases in negative spousal support.
Steine et al., 2020 [35]	n = 506 sexual abuse survivors [Norway] Age:24–47	Data from the Longitudinal Investigation of Sexual Abuse (LISA)	3 waves over 4-year period	Perceived social support [MSPSS]	Anxiety and depression symptoms [HADS]	Cross-lagged panel analyses revealed significant weak reciprocal associations between perceived social support and depression, posttraumatic stress symptoms and anxiety symptoms, but not with insomnia symptoms.
Whitley et al., 2016 [36]	n = 667 African American custodial grandmothers [USA] Age: 33–83	Prospective study	2 timepoints T1: baseline T2: 12-month follow-up	Social support [FSS]	Depression severity [BSI]	Social support was a mediator in the association between depressive symptoms and mental health quality of life for older African American grandmothers (55+); however, this same relationship did not hold for their younger counterparts (≤55).
Zhou et al., 2020 [37]	n = 1137 college freshmen [China] Age: ≥ 18	Panel study	3 waves with 1 month interval between each wave	Perceived Family Support [MSPSS]	Depression severity [PHQ-9]	Family support in Wave 1 decreased compensatory social networking sites (SNS) use for less introverted freshmen in Wave 2 and further decreased depression in Wave 3.
Albuja et al., 2017 [38]	n = 210 women from two clinics that provide prenatal care [Mexico] Age: 20–44	Longitudinal study	T1: 3^rd^ trimester T2: 6 months postpartum	Social Support [PDPI-R social support subscale]	Depressive symptoms [PHQ-9]	Lacking social support during the 3^rd^ trimester of pregnancy was associated with greater depressive symptoms at 6 months in the postpartum, although this relationship depended on the level of endorsement of the traditional female role during pregnancy.
Asselmann et al., 2016 [39]	n = 306 expectant mothers sampled from community in gynecological outpatient settings [Germany] Age: 18–40	Prospective-longitudinal Maternal Anxiety in Relation to Infant Development (MARI) Study	T1: week 10–12 gestation T2: week 22–24 gestation T3: week 35–37 gestation T4: 10 days postpartum T5: 2 months postpartum T6: 4 months postpartum T7: 16 months postpartum	Perceived Social Support [F-SozU K-14]	Maternal depressive and anxiety disorders [CIDI-V]	Perceived social support declined from prepartum to postpartum; levels of prepartum and postpartum social support were lower in women with comorbid anxiety and depressive disorders compared to those with pure depressive disorder(s), pure anxiety disorder(s), or comorbid anxiety and depressive disorders prior to pregnancy.
Asselmann et al., 2020 [40]					Depressive, anxiety, and stress symptoms [DASS-21]	Peripartum depressive, anxiety, and stress symptoms were lower in women with higher perceived social support (b = -0.225 to -0.308).
Cankorur et al., 2015 [41]	n = 730 women from 20 urban and rural antenatal clinics [Turkey] Age: 18–44	Cohort study	T1: 3^rd^trimester T2: 2 months after childbirth T3: 6 months after childbirth	Emotional, practical support [CPQ]	Depressive symptoms [EPDS]	Worse emotional support from mother-in-law was significantly associated with postnatal depression incidence (OR = 0.93, 95% CI 0.87 to 0.99) and worse emotional support from husband with postnatal persistence (OR = 0.89, 95% CI 0.83 to 0.96) of antenatal depression.
Chen et al., 2016 [42]	n = 203 South Asia immigrant mothers [Taiwan] Age: ≥ 18	Panel study	T1: 1 month postpartum T2: 6 months postpartum T3: 1 year postpartum	Emotional, instrumental, informational support [Social Support Scale]	Depressive symptoms [EPDS]	Depression and instrumental support followed downward curvilinear trajectories, while emotional and informational support followed upward curvilinear trajectories. Emotional and instrumental support negatively covaried with postpartum depression over time, but not informational support.
Chen et al., 2020 [43]	n = 407 immigrant and native-born women from obstetrical clinics and hospitals [Taiwan] Age: 20–44	Prospective study	T1: 2^nd^ or 3^rd^ trimester T2: 3 months postpartum	Emotional, instrumental, informational support [Social Support Scale]	Depressive symptoms [EPDS]	Social support was significantly and negatively associated with postpartum depressive symptoms in both immigrant and the native-born women, and presence of depressive symptomatology during pregnancy and a lower level of social support were associated with an increased depressive symptom score at 3 months postpartum.
Faleschini et al., 2020 [44]	n = 1356 women from 8 obstetric offices [USA] Mean age: 32.6	Data from Project Viva, a prospective observational cohort study	T1: trimester visits T1: 6 months postpartum	Perceived social support [Turner Support Scale]	Depressive symptoms [EPDS]	Greater partner support and support from family/friends were strongly associated with lower odds of incident depression (OR 0.33, 95% CI [0.20, 0.55] and OR 0.49, 95% CI [0.30, 0.79]).
Gan et al., 2019 [45]	n = 3310 women from antenatal clinics [China] Age: ≥ 20	Prospective study; Data from Shanghai Birth Cohort	T1: early pregnancy T2: 6 weeks postpartum	Perceived social support [ESSI]	Postpartum depression [EPDS]	Significant associations between low perceived social support and postpartum depressive symptoms were found (Model I odds ratio: 1.63, 95% confidence interval: 1.15, 2.30; Model II odds ratio: 1.77, 95% confidence interval: 1.24–2.52).
Hagaman et al., 2021 [46]	n = 780 women in rural area [Pakistan]	Longitudinal data from the Bachpan Cohort	T1: 3 months postpartum T2: 6 months postpartum T3: 12 months postpartum	Social support [MSPSS, MSSI]	Major depressive disorder [SCID]	High and sustained scores on the MSPSS through the perinatal period were associated with a decreased risk of depression at 12 months postpartum (0.35, 95% CI: 0.19 to 0.63).
Hare, 2020 [47]	n = 144 women at risk for peripartum depression/ euthymic [USA] Age: 19–41	Cohort study	T1 & T2: Twice antepartum T3 & T4: Twice postpartum	Perceived social support [PSSQ]	Peripartum depression [EPDS] Anxiety [HAM-A]	Women diagnosed with PND experienced significantly worse mother–infant bonding and social support compared to HCW (*p* = .001, *p =* .002, respectively) and to those who were at‐risk for but did not develop PND (*p* = .02, *p* = .008).
Hetherington et al., 2018 [48]	n = 3057 women [Canada] Age: ≥ 18	Data from the All Our Families longitudinal pregnancy cohort	T1: 4 months postpartum T2: 1 year postpartum	Support types: tangible, positive social interaction, and emotional/informational support. [MOS-SSS]	Depressive or anxiety symptoms [EPDS]	Low total social support during pregnancy was associated with increased risk of depressive symptoms (RR 1.50, 95% CI 1.24 to 1.82) and anxiety symptoms (RR 1.63, 95% CI 1.38 to 1.93) at 4 months postpartum. Low total social support at 4 months was associated with increased risk of anxiety symptoms (RR 1.65, 95% CI 1.31 to 2.09) at 1 year. Emotional or informational support wan an important type of support for postpartum anxiety.
Leonard et al., 2020 [49]	n = 1316 first time mothers [USA] Age: 18–35	Longitudinal cohort study	5 Time points: 1, 6, 12, 18, and 24 months postpartum	Perceived social support [MOS-SSS]	Maternal postpartum depressive symptoms [EPDS]	Perceived social support decreased, perceived stress increased, and depressive symptoms remained constant from 1 to 18 months, then increased at 24 months. Low perceived social support predicted 6-month depressive symptoms, whereas perceived stress predicted depressive symptoms at all time points.
Li et al., 2017 [50]	n = 240 pregnant women from the prenatal clinic at a general hospital [China] Age: ≥ 18	Longitudinal study	T1: late pregnancy T2: 1 week postpartum T3: 4 weeks postpartum	Perceived social support [MSPSS]	Antepartum depression [EPDS]	Women who had higher Perceived Social Support Scale scores at late pregnancy had less likelihood of developing antepartum depression, and women with higher social support scores at postpartum week 4 were less likely to have postpartum depression. However, the Perceived Social Support Scale scores at late pregnancy did not predict the risk of postpartum depression.
Milgrom et al., 2019 [51]	n = 54 women who met DSM-IV criteria of MDD or minor depression [Australia] Age: ≥ 18	Longitudinal follow-up of a previous RCT for antenatal depression	T1: baseline T2: 9 weeks post-randomization T3: 6 months T4: 9 months T5: 24 months post-birth	Perceived social support [SPS]	Depression [BDI-II] Anxiety [BAI]	Two aspects of social support, reassurance of worth and reliable alliance, were strongly related to perinatal depression and anxiety, particularly when predicting symptoms in late pregnancy. However, the effect of postnatal depression on child development at 9- and 24-months post-birth was not mediated by social support.
Morikawa et al., 2015 [52]	n = 877 women enrolled in a prepartum program during pregnancy [Japan] Age: ≥ 20	Cohort study	T1: before 25^th^ week of gestation T2: 1 month after childbirth	Social support [SSQ]	Postpartum depression [EPDS]	Having a larger number of people available to provide social support during pregnancy has a greater protective effect on pregnant mothers with than without depression.
Nakamura et al., 2020 [53]	n = 12386 couples [France] Age: ≥ 18	Data from the French representative ELFE (Etude Longitudinale Française depuis l’Enfance) cohort study	T1: at birth T2: 2 months post-partum T3: 1year postpartum T4: 2 years post-partum	Informal and formal support [face-to-face and phone interviews]	Parental postnatal depression [EPDS]	Insufficient partner support as well as frequent quarrels during pregnancy predicted the odds of both parents being depressed. This association was higher for women with psychological difficulties during pregnancy than those without. An inverse association was also observed between psychosocial risk assessment attendance (informal support) and joint parental PPD, especially in couples in which the mother had psychological difficulties during pregnancy.
Ohara et al., 2017 [54]	n = 494 pregnant women attending perinatal classes [Japan] Age: ≥ 20	Prospective cohort study	T1: early pregnancy before week 25 T2: 1 month after delivery	Number of persons and satisfaction with social support [SSQ]	Postpartum depression [EPDS]	Satisfaction with the social support received during pregnancy did not directly predict depression in the postpartum period at a statistically significant level. However, poorer satisfaction with the social support received during pregnancy was a cause of depression in the postpartum period due to increased depression during pregnancy.
Ohara et al., 2018 [55]	n = 855 pregnant women attending perinatal classes [Japan] Age: ≥ 20	Cohort study	T1: early pregnancy before week 25 T2: 1 month after delivery	Number of persons and satisfaction with social support [SSQ]	Postpartum depression [EPDS]	Bonding failure in the postpartum period was significantly influenced by mothers’ own perceived rearing as well as social support during pregnancy. In addition, depression in the postpartum period was strongly influenced by social support during pregnancy.
Qu et al., 2021 [56]	n = 66 pregnant women with a history of recurrent miscarriage [China] Mean Age:31.9	Prospective longitudinal study	6–12, 20–24 and 32–36 gestational weeks	Perceived social support [MSPSS]	Anxiety [SAS] Depression [EPDS]	Anxiety and depression were prevalent in pregnant women with a history of recurrent miscarriage, especially in early pregnancy with the lowest level of social support. The correlations among anxiety and social support, and depression and social support at each time point were significant (*p* < 0.05).
Racine et al., 2019 [57]	n = 3388 mothers from community, laboratory, and health care clinic offices [Canada] Age: ≥ 18	Large, population-based cohort All Our Babies/Families (AOB/AOF)	T1: < 25 weeks gestation T2: 34–36 weeks gestation T3: 4 months postpartum T4: 12 months postpartum	Perceived social support: [3 questions]	Prenatal and postpartum anxiety [STAI]	Women who experience heightened stress and anxiety in the perinatal period relative to their own average levels are at risk of higher anxiety and stress at subsequent time points; within-person increases in partner and friend support are salient predictors of subsequent decreases in both stress and anxiety; increases in stress and anxiety in the perinatal period are at risk of experiencing decreases in friend and family support.
Racine et al., 2020 [58]	n = 1994 women from health care and laboratory offices [Canada] Age: ≥ 18	Large, population-based cohort All Our Babies/Families (AOB/AOF)	T1: < 25 weeks gestation T2: 4 months postpartum T2: infant age of 36 months	Maternal social support [MOS-SSS]	Maternal depression [EPDS]	Although maternal social support was a significant predictor of maternal depression across the perinatal period, social support did not moderate the association between ACEs and maternal depression.
Razurel et al., 2015 [59]	n = 235 primiparous mothers [Switzerland] Age:21–43	Longitudinal study	T1: During the last month of pregnancy and T2: 6 weeks after birth	Satisfaction with social support [20-items scale constructed in the area of perinatal care]	Depressive symptoms [EPDS] Anxiety [STAI]	Satisfaction with emotional support in T1 was negatively correlated with depressive symptoms in T1 and T2, which suggested that this type of support was important both in the short and the long term.
Razurel et al., 2017 [60]			T1: gestational weeks 37–41 T2: 2 days post-delivery T3: 6 weeks postpartum			The more the women were provided with support from their partners, the less depressive symptoms and elevated levels of anxiety they reported, even under stressful conditions, while the satisfaction of support from their mothers boosted their sense of competency. Satisfaction with emotional support from professionals tempered the stress during the post-partum period (ΔR^2^ = 0.032; p < .05).
Schwab-Reese et al., 2017 [61]	n = 195 women from a large hospital [USA] Age: ≥ 18	Longitudinal study	T1: following birth T2: 3 months after birth T3: 6 months after birth	Perceived social support [MOS-SSS]	Depressive and anxiety symptoms [DASS–21]	Current perceptions of social support were associated with depressive and anxiety symptoms at three-months postpartum, but social support was not protective against depressive or anxiety symptoms at six-months postpartum.
Senturk et al., 2017 [62]	n = 730 women recruited in their third trimester [Turkey] Age: 18–44	Cohort study	T1: 3^rd^ trimester T2: 0.8–7.4 months after childbirth T3: 10.8–16.6 months T4: 18.1–23.5 months	Quality of relationships and social support [CPQ]	Postpartum depression [EPDS]	Self-rated emotional and practical support from all three relationships worsened over time in the cohort overall. Emotional support from the husband, and emotional and practical support from the mother-in-law declined more strongly in women with depressive symptoms at baseline
Tani et al., 2017 [63]	n = 179 nulliparous pregnant women [Italy] Age: 18–42	Longitudinal study	T1: 31–32 week of pregnancy T2: 1^st^ day after childbirth T3: 1 month after birth	Perceived social support [MSSS]	Postpartum depression [EPDS]	Post-partum depression was influenced negatively by maternal perceived social support and positively by negative clinical birth indices. In addition to these direct effects, analyses revealed a significant effect of maternal perceived social support on post-partum depression, mediated by the clinical indices considered.
Tsai et al., 2016 [64]	n = 1238 pregnant in economically deprived settlements [South Africa] Age: ≥ 18	Population-based longitudinal study	T1: 6 days after birth T2: 6 months post-partum T3: 18 months post-partum T4: 36-month follow-up	Emotional and instrumental support (10 questions about trust and support derived from SOS)	Depression symptom severity [EPDS]	Social support was found to be an effect modifier of the relationship between food insufficiency and depression symptom severity, consistent with the “buffering” hypothesis. Instrumental support provided buffering against the adverse impacts of food insufficiency while emotional support did not.
Yoruk et al., 2020 [65]	n = 317 pregnant women at 38 weeks of gestation [Turkey] Age: 23–34	Longitudinal study	T1: 4th week postpartum T2: 6th week postpartum	Perceived social support [MSPSS]	Postpartum depression [EPDS]	Despite the low level of perceived social support in the group at risk for PPD, this difference was not significant.
Yu et al., 2021 [66]	n = 512 first-time mothers [USA] Mean Age: 21.3	Data from the National Data Archive on Child Abuse and Neglect	T1: 6-month postpartum T2: 12-month postpartum	Social support [SSI]	Depressive symptoms [BDI-II]	Social support was not found to have a direct or indirect effect on postpartum depression/
Zheng et al., 2018 [67]	n = 420 Chinese primiparous women from obstetric wards at hospitals [China] Age: ≥ 18	Longitudinal study	T1: 6 weeks postnatally T2: 12 weeks postnatally	Emotional, material, informational, and evaluation of support [PSSS]	Postnatal depression symptoms [EPDS]	Postnatal depression symptoms and social support are the important influencing factors of maternal self-efficacy. The mean social support scores and scores of emotional support, informational support and evaluation of support had statistically significant increases over time.
Zhong et al., 2018 [68]	n = 3336 women [Peru] Age: 18–49	Pregnancy Outcomes, Maternal and Infant Cohort Study	T1: <16 weeks gestation T2: 26–28 weeks gestation	Satisfaction with social support and number of support providers [SSQ-6]	Depressive symptoms [EPDS]	Low number of support providers at both time points was associated with increased risk of depression (odds ratio = 1.62, 95% confidence interval: 1.12, 2.34). Depression risk was not significantly higher for women who reported high social support at one of the 2 time points.

Checklist: BAI = Beck Anxiety Inventory; PCL-L = Civilian PTSD Checklist; BSI = Brief Symptom Inventory; CES-D = Center for Epidemiological Studies-Depression Scale; CIDI = Composite International Diagnostic Interview; CIS-R = Clinical Interview Schedule–Revised; DASS = Depression Anxiety Stress Scale; DCSQ = Demand-Control-Support-Questionnaire; DRRI = Deployment Risk and Resilience Inventory; FSS = Family Support Scale; GAD = Generalized Anxiety Disorder; HADS = Hospital Anxiety and Depression Scale; IDS-SR = Inventory of Depressive Symptomatology Self-Report version; MINI: Mini-International Neuropsychiatric Interview; MSPSS = Multidimensional Scale of Perceived Social Support; PHQ = Patient Health Questionnaire; SCL-CD6 = Symptom Checklist Core Depression Scale; SIAS = Social Interaction Anxiety Scale; SPS = Social Provisions Scale.

Checklist: BAI = Beck Anxiety Inventory; BDI = Beck Depression Inventory; CIDI = Composite International Diagnostic Interview for Women; CPQ = Close Persons Questionnaire DASS = Depression Anxiety Stress Scale; EPDS = Edinburgh Postnatal Depression Scale; ESSI = ENRICHD Social Support Instrument; F-SozU K-14 = Brief form of the Perceived Social Support Questionnaire; HAM-A = Hamilton Anxiety Rating Scale; MOS-SSS = Medical Outcomes Study Social Support Survey; MSPSS = Multidimensional Scale of Perceived Social Support; MSSI = Maternal Social Support Index; MSSS = Maternal Social Support Scale; PDPI-R = Postpartum Depression Predictors Inventory-Revised; PHQ = Patient Health Questionnaire; PSSQ = Postpartum Social Support Questionnaire; PSSS = Postpartum Social Support Scale; SCID = Structured Clinical Interview for DSM Disorders; SAS = Self-Rating Anxiety Scale; SOS = Significant Others Scale; SPS = Social Provisions Scale; SSI = Social Support Interview; SSQ = Social Support Questionnaire; STAI = Spielberger State Anxiety Scale.

**Table 2:** Characteristics of included studies on social isolation.

Reference	Sample/Setting [Country]	Study Type	Follow-up Times	Social Isolation [Measure]	MH Outcome [Measure]	Main Findings for Social Isolation
Domènech-Abella et al., 2019 [69]	n = 5066 adults [Ireland] age ≥50	Irish Longitudinal Study on Ageing (TILDA)	2 waves of TILDA Second wave: 2012–13 Third wave: 2014–15	UCLA Loneliness Scale	Major depressive disorder (MDD) or generalized anxiety disorder (GAD) [CIDI]	The longitudinal association between experiencing loneliness and higher likelihood of suffering from GAD two years later is bidirectional, whereas the association between social isolation and higher likelihood of subsequent MDD or GAD as well as those between loneliness and subsequent MDD or deterioration of social integration are unidirectional.
Domènech-Abella et al., 2021 [70]	n = 895 older adults [Netherlands] Age: ≥75	Longitudinal Aging Study Amsterdam (LASA)	4 waves over 10 years	De Jong Gierveld Loneliness scale	Depressive symptoms [CES-D]	Loneliness was positively associated with depressive symptomatology, but not vice versa.
Evans et al., 2019 [71]	n = 2135 elderly residents [Wales] Age: ≥65	Data from the Cognitive Function and Ageing Study–Wales (CFAS-Wales)	2 timepoints T1: baseline T2: 2-year follow-up	LSNS-6	Depression and anxiety [AGECAT]	Older people with depression or anxiety perceived themselves as more isolated than those without depression or anxiety, despite having an equivalent level of social contact with friends and family. In people with depression or anxiety, social isolation was associated with poor cognitive function at baseline, but not with cognitive change at 2-year follow-up.
Förster et al., 2021 [72]	n = 679 elderly individuals [Germany] Age: 80+	Longitudinal study AgeCoDe and its follow-up study AgeQualiDe	Data from follow-up 5 to follow-up 9 (2011–2016)	LSNS-6	Depression [GDS-15]	“Widowed oldest old”, who are also at risk of social isolation, reported significantly more depressive symptoms in comparison to those without risk.
Herbolsheimer et al., 2018 [73]	n = 334 community-dwelling older adults [Germany] Age: 65–84	Longitudinal study	2 timepoints T1: baseline T2: 3 years later	LSNS-6	Depressive symptoms [HADS]	Being socially isolated was associated with lower levels of out-of-home physical activity, and this predicted more depressive symptoms after 3 years. However, no direct relationship was observed between social isolation from friends and neighbors at the baseline and depressive symptoms 3 years later.
Holvast et al., 2015 [74]	n = 285 older adults [Netherlands] Age: ≥ 60	Multi-site prospective cohort study from the Netherlands Study of Depression in Older Persons (NESDO)	2 timepoints T1: baseline T2: 2-year follow-up	De Jong Gierveld Loneliness scale	Depression [CIDI] Depression severity [IDS-SR]	Loneliness, subjective appraisal of social isolation, was a significant positive determinant of depressive symptom severity during follow-up. This association was independent of social network size and persisted after controlling for other potential confounders.
Lee et al., 2021 [75]	n = 4211 adults [UK] Age: ≥ 50	Data from English Longitudinal Study of Ageing (ELSA)	7 waves collected once every 2 years between 2004 and 2017	R-UCLA	Depressive symptoms [CES-D]	Loneliness, irrespective of other social experiences, was associated with a heightened risk of depression, and this risk persisted for up to 12 years after the loneliness was reported.
Martín-María et al., 2021 [76]	n = 1190 older Spanish adults Age:50+ [European countries]	Longitudinal study	Interviewed on 3 evaluations over a 7-year period	UCLA Loneliness Scale	Depression [CIDI]	Participants experiencing chronic loneliness were at a higher risk of presenting major depression (OR = 6.11; 95% CI = 2.62, 14.22) relative to those presenting transient loneliness (OR = 2.22; 95% CI = 1.19, 4.14).
Noguchi et al., 2021 [77]	n = 3331 respondents [England and Japan] Age: ≥ 65	Data from English Longitudinal Study of Ageing (ELSA) and Japan Gerontological Evaluation Study (JAGES)	Followed up regarding depression onset for 2 years (2010/2011–2012/2013) for ELSA and 2.5 years (2010/2011–2013) for JAGES	Modified version of SSI	Depressive symptoms [CES-D. GDS-15]	Social isolation was significantly associated with depression onset in both England and Japan, despite variations in cultural background.

Checklist: AGECAT = Automated Geriatric Examination; CIDI = Composite International Diagnostic Interview; GDS = Geriatric Depression Scale; HADS = Hospital Anxiety and Depression Scale; IDS-SR = Inventory of Depressive Symptomatology Self-Report version; LSNS = Lubben Social Network Scale; R-UCLA = Revision of the University of California, Los Angeles Loneliness Scale; SSI = Social Isolation Index.

**Table 3:** Characteristics of included studies on social network.

Reference	Sample/Setting [Country]	Study Type	Follow-up Times	Social Network [Measure]	MH Outcome [Measure]	Main Findings for Social Network
Baek et al., 2021 [78]	n = 291 married couples [South Korea] Age: ≥60	Korean Social Life, Health, and Aging Project (KSHAP)	5 waves: 2011, 2012, 2014–15, 2015–16, 2018–19	Fours questions in total about supportive relations and negative relations	Depressive symptoms [CES-D]	The association between husbands’ and wives’ depressive symptoms was stronger for couples that reported a low level of supportive marital relations, but only for the wife and those that reported a high level of negative marital relations for both the husband and wife.
Chang et al., 2016 [79]	n = 21728 elderly women [USA] Age: ≥65	Prospective cohort study: Nurses’ Health Study (NHS)	5 timepoints, Baseline and 4 biennial follow-up questionnaire cycles over 10-year period	Berkman-Syme Social Network Index	Depression [MHI-5 subscale of the SF-36, CESD-10, GDS-15]	Social factors (lower social network; lower subjective social status; high caregiving burden to disabled/ill relatives) were associated with higher incident late-life depression risk in age-adjusted models.
Domènech-Abella et al., 2021 [70]	n = 5066 older adults [Ireland] Age: ≥75	Longitudinal Aging Study Amsterdam (LASA)	4 waves over 10 years	Names of persons with whom they had regular contacts in the past year	Depressive symptoms [CES-D]	Decreasing social network size (Coef. = -0.02; p < 0.05), predicted higher levels of loneliness, which predicted an increase in depressive symptoms (Coef. = 0.17; p < 0.05) and further reduction of social network (Coef. = -0.20; p < 0.05).
Domènech-Abella et al., 2019 [69]	n = 5066 adults [Ireland] Age: ≥50	Irish Longitudinal Study on Ageing (TILDA)	2 waves of TILDA Second wave: 2012–13 Third wave: 2014–15	Berkman-Syme Social Network Index	MDD or GAD [CIDI]	Both objective social isolation (size of social network) and loneliness factors have been found to be robust risk factors for depression and anxiety independently, which acts as a warning not to underestimate the subjective aspects of social isolation.
Förster et al., 2018 [80]	n = 783 elderly people [Germany] Age: ≥ 75	Population-based cohort study: Leipzig Longitudinal Study of the Aged (LEILA)	3 timepoints T1: baseline T2: follow-up1 T3: follow-up2	PANT	Depressive symptoms [CES-D]	Persons with a restricted social network were more likely to develop depression, and risk of depression was particularly high for elderly with social loss experiences.
Noteboom et al., 2016 [29]	n = 1085 respondents from health care settings [Netherlands] Age:> 18	Longitudinal cohort study: Netherlands Study of Depression and Anxiety (NESDA)	2 timepoints T1: baseline T2: 2-year follow-up	Questions on how many relatives, friends or others over the age of 18 years they had regular and important contact	Depressive disorders [CIDI]	Structural (network size and partner status) did not predict depression at follow up. Pariticipants with a lifetime history of depression reported a smaller social network.
Van Den Brink et al., 2018 [30]	n = 1474 patients with a major depressive disorder [Netherlands] Age:18–90	Data from 2 cohort studies NESDA and Netherlands Study of Depression in Older Persons (NESDO)		Questions on social network characteristics	Presence of depression [CIDI] Depression severity [IDS-SR]	Social network characteristics, such as having a partner and number of persons in one’s household, are related to depression course.
Reynolds et al., 2020 [81]	n = 3005 elderly people [USA] Age:57–85	Panel data from National Social Life, Health, and Aging Project (NSHAP)	3 waves 2005, 2010, and 2015 with 5-year intervals between each wave	Questions on community-layer, interpersonal-layer, and partner-layer connection	Depressive symptoms [CES-D]	Results demonstrate multiple links between social connection and depression, and that the evolution of social networks in older adults is complex, with distinct mechanisms leading to positive and negative outcomes. Specifically, community involvement showed consistent benefits in reducing depression.
Santini et al., 2020 [82]				Social Disconnectedness Scale	Depressive symptoms [CES-D-ML] Anxiety [HADS-A]	Perceived isolation was positively associated with depression symptoms at T2 and T3 (β = 0·12; p<0·0001).
Santini et al., 2021 [83]	n = 38300 adults [13 European countries] Age: ≥ 50	Survey of Health, Ageing and Retirement in Europe (SHARE)	2 consecutive waves (2011, 2013) of the SHARE survey	The total number of close relations in the social network	Depressive symptoms [EURO-D Scale]	Social participation among people with relatively few close social ties was negatively associated with depression symptoms but did not seem to benefit to those with relatively many close social ties.

Checklist: CES-D = Center for Epidemiological Studies-Depression Scale; CES-D-ML = Center for Epidemiological Studies-Depression Minus Loneliness Scale; CIDI = Composite International Diagnostic Interview; GAD = Generalized Anxiety Disorder; GDS = Geriatric Depression Scale; HADS = Hospital Anxiety and Depression Scale; IDS-SR = Inventory of Depressive Symptomatology Self-Report version; MDD = Major Depressive Disorder; MHI-5 = Mental Health Index-5.

#### Social support

A little over half of the recent articles on the effect of social support on depression addressed the issues of depression in pregnant women, while the rest addressed the association of social support with anxiety or depression, in a variety of populations, other than pregnant women. We begin our review with nonpregnant populations because the results are of broader general relevance.

*Study Samples other than pregnant women*. Table 1A lists the 24 articles that reported quantitatively on the longitudinal effects of social support on depression or anxiety in samples other than pregnant women, but two papers reported on one study, resulting in a total of 23 studies. The sample size of the included studies ranged between 86 and 15,105 participants. Majority of the studies were conducted in North America (11 studies), and the remaining were from Europe (7 studies), Asia (2 studies), and Australia (2 studies). One study was international in scope and sampled students from 76 host countries.

*Assessing depression/Anxiety and social support*. The twenty-three included studies used a threshold score on a depression rating scale to measure depressive symptoms, and the Center for Epidemiological Studies Depression Scale was the most frequently utilized scale, being used in eight studies. Other measures with established psychometric properties included in this Table were as follows: Patient Health Questionnaire‐9, Geriatric Depression Scale, Inventory of Depressive Symptomatology, Symptom Checklist Core Depression Scale, and Brief Symptom Inventory. In order to assess anxiety and depressive symptoms, three studies [17,19,35] used the Hospital Anxiety and Depression Scale, and two [21,31] used Depression Anxiety Stress Scale. Furthermore, some studies employed [29,30,33] structured diagnostic interviews for common mental disorders. The social support measures in these studies varied considerably, with five studies [15,16,26,31,35,37] using the original or the adapted version of the Multidimensional Scale of Perceived Social Support (MSPSS). Two studies [14,17] employed Demand-Control-Support-Questionnaire (DCSQ) to assess workplace social support, and three studies [18,19,32] used Social Provision Scale (SPS). A scale in the Deployment Risk and Resilience Inventory (DRRI) was employed in one veteran study to assess the extent to which they perceive assistance and encouragement in the war zone from fellow unit members [21]. Five studies [20,22,27,28,34] used questionnaires that were developed by the authors. The Interview Schedule for Social Interaction—availability of Attachment Scale was used to measure the perceived availability of interpersonal support [24]. Whitley et al [36] used the Family Support Scale (FSS), and Haverfield et al. [25] used a subscale of the Basic Need Satisfaction Scale to assess general social support (Table 1A).

*Effects on depression/Anxiety*. Social support has been shown benefit in abating symptoms of depression over time in 19/23 or 82.6% of the studies (Table 1A). Analyses of mental health conditions in a sample of nationally dispersed war-zone veterans for over seven years indicated that higher levels of social support post-deployment were associated with decreased risk of depression and anxiety disorders, as well as less severe symptoms [21]. Moreover, reduced levels of perceived support at the workplace were associated with increased levels of depression symptoms [14], which aligns with previous research that showed a direct influence of social support on the well-being of medical staff workers in the subsequent study waves [17]. Apart from these direct effects, social support has also been shown to have "buffering" effects on the association between involuntary job loss and depressive symptoms among a subgroup of persons who are more likely to be White, educated, and have high levels of social support before becoming unemployed [20]. In a longitudinal study among emergency medical care providers conducted in the United States by Feldman and colleagues [23], social support at baseline was identified as a predictor of post-traumatic stress disorders (PTSD), depression, and anxiety symptoms at 3-month follow-up.

A five-year study of rural community residents also found that low perceived interpersonal support was associated with adverse mental health outcomes, including depression [24]. The impact of social capital in the social support dimension on psychiatric health may be differentiated by gender [33]. In a multicenter cohort of civil servants, Souto et al. [33] reported an association between social support and the maintenance of depressive episodes among women (RR = 2.66; 95% CI: 1.61–4.41). However, among men, the authors found an inverse relationship between social capital in the “social support” dimension and incidence of depressive episodes (RR = 1.66; 95% CI: 1.01–2.72) [33]. Billedo et al. [18] found short-term reciprocal associations between social support and depressive symptoms in sojourning students. They further stated that face-to-face interaction with the host-country network had immediate positive effects on perceived social support, which subsequently predicted lower depressive symptoms [18]. Even in samples of emerging adults, higher levels of perceived social support were protective against depressive and anxiety symptoms [22,32]. Boyden and colleagues followed a sample of parents of critically ill children over two years and found that greater perceived social support was associated with lower anxiety levels across assessments [19]. Higher baseline social support remained negatively associated with lower parental anxiety scores at 12 months (B = -0.12, *p* = 0.03; 95% CI = -.23 to -.01) and 24 months (B = -0.11, *p* = 0.04; 95% CI = -0.21 to -0.01). This inverse association had dissipated by 24 months in their adjusted modeling [19]. Their findings concur with other aforementioned studies that demonstrated a benefit of having supportive relationships.

Interestingly, in terms of the source of social support, the role of family support remains unclear. One study of married Arab immigrant women in the U.S. did not find family support to be protective against depression. Instead, support from friends was found predictive of fewer depressive symptoms at follow-up [16]. However, the results of this study contrast with those of Zhou et al., [37] who showed that more perceived family support decreased usage of social networking sites, which was followed by decreased depressive symptoms among Chinese college students. Similarly, Haverfield et al. [25], analyzed data from patients with co-occurring disorders at treatment intake and across follow-ups in the United States and found that deficits in family support were the most consistent predictor of greater depression and substance use severity [25]. Consistent with previous studies on social support drawn from family and their positive impacts, two studies [26,36] specifically focused on custodial grandparents in the U.S. showed that while elevated caregiving stress may negatively affect grandparent caregivers’ mental and overall health over time, greater social support from family networks may reduce depression accompanying caregiving. According to Whitley et al., [36] social support has a mediating effect on the relationship between depression and mental health quality of life in older (55+) African American custodial grandmothers, but not in their younger (≤55) counterparts.

Although depression peaks in young adulthood, it either can persist or emerge later in life, as evidenced by the three studies focusing exclusively on the role of social support in samples of adults aged 55 and older. Misawa and Kondo (2019), in a study of 3464 Japanese older people, reported that social support was unrelated to changes in depressive symptoms but added that social factors of having hobbies and meeting frequently with friends were associated with improved late-life depression. This link between social interaction and social support was only observed to be protective for men [28]. Conversely, another study spanning eight years of follow-up of older adults in England found a bidirectional association between depressive symptoms and spousal support [34]. The authors found an average decrease in positive support (or an increase in negative support) over time in age- and gender-adjusted models, which later predicted increasing depressive symptom trajectory [34]. Another study on older people with depression revealed that a chronic course of depression might decrease received social support over time [27]. Moreover, their findings suggest that pre-existing depression in concert with less social support may predispose older persons, especially men and single people, to more depression over time [27].

A few studies (3/19 = 16%) found minimal or no evidence that lower levels of social support predict depression at follow-up. For example, in a naturalistic cohort study of Noteboom et al. [29], people with a prior history of depression reported a smaller network size and less emotional support at baseline However, these structural (network size or having a partner) and the perceived aspects of social support had no predictive value in the longitudinal data. Similarly, only negative experiences with social support proved to be a risk factor for non-remission, independent of other social-relational variables in depressed persons [30]. Steine et al. [35] found statistically significant weak reciprocal associations between perceived social support and depression and anxiety symptoms over time among adult survivors of childhood sexual abuse. Although Porter and Chambless reported a link between higher odds of relationship dissolution and social anxiety, they observed no differences between participants with high versus low social anxiety with regard to the amount of social support provided by their partners [31].

*Social support for women during and post pregnancy*. Table 1B presents results from thirty-one articles examining associations between social support and depression during pregnancy or postpartum, uniquely vulnerable periods for women during which they may experience a range of psychosocial stressors. Two pairs of papers provided findings based on identical samples and were reported in a combined table entry, resulting in twenty-nine studies. Studies were predominantly conducted in Asia (45%) and North America (32%), with the remaining studies from Europe (14%), South America (3%), Africa (3%), and Australia (3%). In terms of individual countries, six studies were conducted in the United States, followed by four studies in China, and three studies each in Canada, Japan, and Turkey. Sample sizes varied from 54 participants in a follow-up of a previous randomized trial [51] to 12,386 couples in a study with findings on both maternal and paternal depression [53]. Most of the included studies [38–41,43,44,47,50,51,58–60,62,63,68] in the mid or late pregnancy, four [45,54–56] started in early pregnancy, and ten studies [42,46,48,49,53,61,64,65,67,70] started after birth. Women were on average aged between 18 and 40 years old. The most commonly used measures of social support and depression were, respectively, the Medical Outcomes Study Social Support Survey (MOS-SSS) and the Edinburgh Postnatal Depression Scale (EPDS) (Table 1B).

The majority of the studies (24/29 = 83%) found low social support increased postpartum depressive symptoms. Women with higher perceived social support exhibited lower depression (b = -0.308, SE = 0.036, p < .001) and anxiety (b = -0.225, SE = 0.039, *p* < .001) symptom severity across the peripartum period [40]. Similar results were obtained by other studies [45,48,51,58,68] conducted to investigate the trajectory of the association between depression and perceived social support. A study in Taiwan comprising 407 immigrant and native-born women showed that a presence of depression symptoms during pregnancy (β = 0.246; *p*<0.001) and deficient social support (β = -0.233; p<0.001) positively covaried with depressive symptoms at 3- months postpartum [43]. The significant protective factor of social support against postpartum depression was also highlighted in another study through a mediating effect of good clinical delivery.^46^ Hagaman et al. [46] employed multiple time-varying measures of social support to evaluate the causal effect of longitudinal patterns on perinatal depression outcomes at six and twelve months postpartum. The authors found that women who had sustained high scores on the MSPSS had reduced prevalence of depression at one-year post-partum [46]. Moreover, insufficient social support and frequent quarrels during pregnancy were associated with significant increase in joint postpartum depressive symptoms in mothers and fathers [53]. Tani et al. demonstrated a protective role of maternal and paternal relationships on postpartum depression in nulliparous women [63].

Asselmann and colleagues [39] found a bidirectional relationship between peripartum social support and psychopathology. They concluded that low social support increased the risk for anxiety and depressive disorders, and these disorders before pregnancy, in turn, fostered dysfunctional relationships and lowered social support across the peripartum period [39]. Women with comorbid anxiety and depression were at higher risk for lacking social support during this timeframe [39]. Social support appeared to be a predictor of depression from mid-pregnancy to six months postpartum, particularly in late pregnancy. Nonetheless, the predictive effect of social support on anxiety was only observed in late pregnancy [68]. Latina women who had lower social support during the third trimester of pregnancy were reported to be at greater risk of depressive symptoms at six months in the postpartum, which was consistent with a study on primiparous mothers [49]. Specifically, we found that women with lower social support and higher self-reported adherence to the Traditional Female Role were at the highest risk of experiencing postpartum depressive symptoms [38]. A study conducted in China found that anxiety and depression are highly prevalent in pregnant women who have experienced recurrent miscarriages, particularly in the early weeks of pregnancy when social support is at its lowest level [56]. These findings converge with those from the past studies that identified social support as an important buffer against anxiety and depression throughout the pregnancy [56].

Data from Canada reported inverse associations between 1) social support during pregnancy and anxiety and depression postnatally (RR 1.50, 95% CI 1.24 to 1.82) and 2) social support at four months postpartum and one year postpartum (RR 1.65, 95% CI 1.31 to 2.09) [48]. In contrast to these studies, Schwab-Reese et al. stated that social support was not protective against depressive or anxiety symptoms at six months postpartum as it was at three months postpartum [61].

Satisfaction with all the types of support (emotional, material, esteem, and informative) from the spouse reduced the psychological disorders in mothers as much in the prenatal compared to the postpartum period [59]. Among immigrant mothers in Taiwan from China or Vietnam, emotional support was found to be significantly and inversely associated with postpartum depression [43]. Emotional and informational support were identified as the most important types of social support for postpartum anxiety [48]. Marginal structural models were employed to evaluate the time-varying associations of low social support (before and in early pregnancy) with depression in late pregnancy in a cohort of Peruvian women, and analyses suggested that women with sustained low scores on the 6-item Sarason Social Support Questionnaire (SSQ-6) were at higher risk of antepartum depression [68]. The authors also found a stronger association of fewer persons providing social support on depression risk than low social support satisfaction.

Others have found that not all forms of social support during a woman’s transition into motherhood are equally beneficial in alleviating depression and anxiety symptoms. Razurel et al. reported that support provided by one’s partner buffered the effects of stress on depression, although support from friends or professionals did not [60]. Although social support from family and friends was deemed less prominent than that from the spouse, studies [44,59] showed that these sources of support also had an influence on maternal mental health. A study evaluating the impact of family relationships and support on perinatal depressive symptoms between the third trimester of pregnancy and two to six months postpartum noted that the incidence and persistence of depression symptoms were predicted by lower baseline perceived emotional support from the mother-in-law and the husband, respectively [41]. In a large population-based study, increases in partner and family support had a more protective effect against anxiety and stress, and higher than average levels of anxiety and stress led to maternal-reported decreases in support [57].

Some studies [50,65,66] did not find associations between low perceived social support during pregnancy and postpartum depression. Ohara et al. [54] showed that satisfaction with social support did not directly predict depression in the postpartum period at a statistically significant level. Further, their path model revealed that less satisfaction with the social support received during pregnancy was rather a cause of postpartum depression. This indirect link of social support with depression in the postpartum period is in line with another study with slightly larger sample size, one year later, by the same first author [55]. While one study [52] found that the number of supportive persons during pregnancy had a more substantial effect on decreasing postpartum depressive symptoms in depressed relative to non-depressed mothers, their analyses also showed that satisfaction with social support was not a significant predictor of postpartum depression. The inconsistent findings on social support and depression and anxiety across included studies can be partially attributed to varying operational definitions of social support, utilization of different self-report social support and depression measures, and insufficient control for confounding variables.

#### Social isolation/Loneliness

Given that both these concepts were concurrently assessed in addressing mental health outcomes in some studies, articles that met the inclusion criteria of the current review and examined the subjective counterpart of social isolation were included in Table 2. This table presents characteristics of selected studies that explored the impact of social isolation on depression and anxiety disorders. The included studies reported on a total of 22,976 older adults aged 50 and above in European countries and Japan. The number of participants in the studies varied considerably, from 285 participants with a primary diagnosis of depression in a two-year follow-up study to 5,066 in a report on a well-characterized cohort of adults from Ireland [69]. Social isolation and loneliness (perceived social isolation) are intricately related, albeit conceptually distinct.

All these studies have identified social isolation and/or loneliness as a potential risk factor for depression and anxiety among older adults [69–77]. Domènech-Abella and colleagues evaluated data from the Irish Longitudinal Study on Ageing and Longitudinal Aging Study Amsterdam, in Ireland and the Netherlands, respectively. Social isolation and loneliness were identified as antecedent risk factors for incident depression or exacerbating late-life generalized anxiety or major depressive disorder independently [69]. The authors emphasized the need to address the subjective aspects of social isolation through interventions aimed at improving the characteristics of middle-aged and older adults’ social environments in order to improve their mental health.[69,70]. Holvast et al. [74] found that loneliness was independently associated with more severe depressive symptoms at follow-up (*β* = 0.61; 95% CI 0.12–1.11). These findings are in line with those of a cohort study of adults aged 50 and older, that found that loneliness was linked to depression or increased depressive symptoms, irrespective of objective social isolation, social support, or other potential confounders such as polygenic risk profiles, and that this association persisted 12 years after loneliness had been reported [75]. Similar effects were observed in a cross-national longitudinal study that assessed the relationship between social isolation and depression onset in England and Japan [77], as well as in a study of German primary care sample of the oldest old, those aged 80 years and over [72]. Elderly people who have lost a spouse are at higher risk, as insufficient social network may increase social isolation, potentially contributing to subsequent depressive symptoms [72]. Even after adjusting for the effect of widowhood and other confounders, Martín-María and colleagues [76] found both types of loneliness (i.e., transient and chronic) to be significantly associated with depression. Another study found that participants who scored high on the subjective appraisal of social isolation, measured by six-item de Jong Gierveld Loneliness Scale with subscales for emotional loneliness (perceived absence of intimate relationships) and social loneliness (perceived lack of a wider circle of friends and acquaintances), at baseline had a lower likelihood of achieving remission two years later [74].Three studies [71–73] utilized the Lubben Social Network Scale, a validated instrument designed to gauge social isolation in the elderly.

Analysis of Cognitive Function and Ageing Study-Wales (CFAS-Wales) by Evans et al. (2019) showed that people with depression or anxiety experience poorer social relationships and higher social isolation and loneliness relative to those without such symptomatology, despite reporting an equivalent level of social contact [71]. The apparent reductions in negative affect at follow-up in over half of the respondents with clinically relevant depression or anxiety, diagnosed using the Automated Geriatric Examination for Computer-Assisted Taxonomy (AGECAT) algorithm, at baseline could be due to biopsychosocial changes intrinsic to ageing and/or cohort effects. Results by Herbolsheimer et al. (2018) opposed previous findings, stating that social isolation from friends and neighbors at the baseline was not directly associated with depressive symptoms at the 3-year follow-up [73]. Of importance, the authors also showed that being socially isolated from friends and neighbors was related to lower levels of out-of-home physical activity, which predicted more depressive symptoms after three years (*β* = .014, 95% CI .002 to .039) [73]. These reported findings demonstrate the potentially detrimental effect of objective and subjective social isolation on mood disorders in later life. Extant evidence suggests the importance of considering both social isolation and loneliness without the exclusion of the other in efforts to mitigate risk.

### Social networks

Eight studies, i.e., ten papers, were identified that examined the longitudinal relationship between social networks and depression or anxiety (see Table 3). Seven [69,70,78–83] of the eight included studies on social networks focused on older adults. Community-dwelling individuals aged 75 and older with restricted social networks were more likely to develop depression compared to those who maintained an integrated social network [80]. While it has also been noted that respondents who experienced social loss within the last six months reported a higher risk of depression in old age, the adverse effects of loss on depression could be attenuated by the existence of an integrated social network [80]. According to a study conducted among Korean couples aged 60 years and older, spousal network overlap was associated with less depression concordance for husbands, however not for wives, which indicates the need for gender-specific strategies to support psychological well-being of older adults. [78]. In a large follow-up study spanning ten years that recruited older female nurses, lower social networks increased the risk of incident late-life depression in age-adjusted models [79]. In line with these results, another study on the elderly showed that a smaller network size measured using the Berkman-Syme Social Network Index is a robust risk factor of major depression and generalized anxiety disorder [69]. Conversely, one study on American adults aged 57–85 years denies this relationship as no direct effects of social networks on frequencies of depression symptoms were detected at follow-up [82]. It is worth noting that the same study also showed that social networks predicted higher levels of perceived isolation (β = 0·09; p<0·0001), which in turn predicted higher levels of depression and anxiety symptoms (β = 0·12; p<0·0001) [82]. According to papers [29,30] that used data from NESDA and NESDO, the size of social networks at baseline did not predict depression at follow-up. For adults with a pre-existing diagnosis of major depressive disorder, only the social network characteristic of living in a larger household was reported to have a unique predictive value for depression course [30].

While smaller social networks emerged as a predictor of depression in three of the five studies, the remaining studies showed no predictive value of social networks in the depression course (3/8 = 37.5%). Santini et al. [83] reported that formal social participation (i.e., active involvement in volunteer organizations, educational institutions, clubs, etc.) was negatively associated with depressive symptoms among individuals reporting few close social ties, but not for those with seven or more close social ties. Instead, individuals with numerous social ties reported an increase in depressive symptoms [83]. The extent to which measures assessed the concept of social networks across the included studies could be disputed and may account for between-study inconsistencies. Some assessed using a count of the number of people in the social network [29,30,83], one assessed social network type (restricted vs. integrated) [80], four assessed network size and frequency of interaction with network members [69,70,78,79,81,82], and one study assessed additional indicators such as three layers of human relationships (community-layer, interpersonal-layer, and partner-layer connection) [81]. These longitudinal studies found that social networks have both direct and buffering effects on psychological wellbeing.

## Discussion

The goal of this scoping review was to determine current knowledge regarding the effect of social connectedness on depression and/or anxiety symptoms and diagnoses in the general population. Lund et al. (2018) noted that while there is no universal definition of social connectedness, it is considered an umbrella term that generally includes the following components: social support, social networks, absence of social isolation and loneliness [84].

Much of the research investigating the role of social connectedness in the studies published in the last five years has focused primarily on social support as compared to the other components of social connectedness and, more specifically, on perceived emotional support. The majority of studies, regardless of whether the support was received or perceived, showed that, in general, social support was a protective factor for both depressive symptoms and disorders. The consistency of the findings across a variety of settings, measures of exposure, and populations supports the generalizability of the findings in this review. The measures used varied in the type of social support assessed, although most studies assessed subjective and objective emotional support rather than structural support.

Few of the studies examined the effect of social support on anxiety symptoms or diagnoses, and those that did investigate these associations found the results mixed and weak. A single study examined the effect of social support on social anxiety and found no association [31]. However, there were too few studies to draw any firm conclusions. Over half of these studies investigated the effects of social support on depression/ anxiety at various stages of pregnancy. The preponderance of studies that investigated the effect of social support on pre/postpartum depression could be due to our restriction of included studies in this review to longitudinal studies since the effects of social support during pregnancy on postpartum depression can readily be assessed by employing longitudinal study design.

There were far fewer studies that examined the longitudinal relationship between social networks and anxiety or depression, the majority of which focused on older adults. Although measures which assessed the concept of social networks were somewhat inconsistent [69,70,78–83] and may have led to mixed results between studies, on the whole, social networks have direct and buffering effects on depression /anxiety. More studies of the effects of social networks such as size and structure and the characteristics of network ties, frequency of contact, reciprocity, duration, and intimacy will give us a greater understanding by which these networks impact depression and anxiety, and opportunities for intervention.

A small number of studies met our inclusion criteria for exploring the impact of social isolation on depression and anxiety. Despite some methodological limitations of these studies, there was fairly strong evidence that both social isolation and loneliness (perceived social isolation) were potentially detrimental in terms of development of mood disorders in later life.

The extant literature recommends that the effects of the individual components rather than a composite measure of social connectedness be used because the correlation between these components is relatively low. Investigating the effects of individual components could lead to greater insight into possible causal pathways and appropriate interventions. Epidemiological research has generally focused on the structural (e.g., social network size /density, marital status, living arrangements) [24,25,30] or functional aspects of social relationships (received and perceived social support, perceived social isolation) [39,40,51], while some researchers have also studied multi-dimensional approaches, i.e., a combination of structural and functional aspects [43,85]. On the rare occasions where researchers have attempted to combine the effects of the components of social connectedness on the diagnostic outcomes, two different approaches have been used: 1. They have either used multivariable regression models (linear or logistic–depending on the outcome) with each of the components included as a separate predictor [43]. They have treated “social connection” as an underlying latent variable, while treating the individual components as the observed or “manifest” variables [85].

Inferences from this review should be drawn cautiously, considering some limitations. Firstly, we only included longitudinal studies published in the English language. Other papers may be available in different languages. Secondly, we conducted a scoping review and not a systematic review; thus, the included papers were not assessed based on their quality. Some of the included studies in this review have small sample size [18,26,47,51,61,63]. These studies must be replicated in larger, heterogeneous populations with a longer interval between the baseline assessment of social connectedness measures and follow-up of depressive and anxiety symptoms to ensure reliable and valid results for inferring the direction of causality. Despite these limitations, the longitudinal/prospective research reviewed here provides current knowledge on the effects of social connectedness on depression and anxiety and sheds important insights into possible causal mechanisms and a deeper understanding of how prior social circumstances can affect later mental health outcomes.

Social support may be interpreted, perceived, and even experienced differently depending on the surrounding sociocultural environments. Cultural differences exist in individuals’ willingness to seek and use social support as a coping mechanism for stressors [86]. Thus, developing culturally valid measurement scales will enable future research to examine the various types and sources of social connectedness across cultures. It is often assumed that the conventional Western typologies and measures developed in individualistic cultures can be applied universally. These assumptions, however, fail to take into consideration the differences in individualistic and collective cultures. While patterns of social support varied with ethnicity and culture, there was no consistent patterns. For instance, while women of Arab origin drew increased emotional support with friends, women of Chinese origin were found to draw emotional support from spouses etc. Larger samples and more homogenous samples are needed to identify more specific patterns. There is a renewed interest in social connectedness as protective of mental health, broadly, and suicidal thoughts and ideas more specifically, as witnessed by the interest shown by NIMH in their call for RFPs to determine the effect of social connectedness on suicidal thoughts and ideation.

### Clinical and policy implications

The results of this scoping review have implications for clinical practice. Strong associations between lacking social connectedness and risk of depression underscore the importance of improving clinical detection of high-risk patients, including those with low received or perceived social support and elevated perceived loneliness. Because these characteristics may be difficult for primary care physicians to detect in their patients [87], consideration might be given to implementing brief screening instruments analogous to clinical tools that have been recommended to assess network size [88]. The link between social connectedness and improved adherence to medical recommendations [89] may motivate practitioners to detect and seek to address social isolation in their patients. However, meaningful clinical progress in addressing a lack of social connectedness may depend upon readily accessible and effective interventions.

The results also have implications for social policy. Because the prevalence of loneliness increases [90] and the size of social networks declines [91] as older adults age, the present findings bolster the public health rationale for addressing social vulnerabilities among older adults. Although some aspects of loneliness and social isolation likely involve genetic susceptibility [92], other aspects appear to involve characteristics of the neighborhood environment. Policymakers seeking to reduce the adverse mental health effects of social isolation among older adults, for example, might weigh the benefits of designing supportive housing structures and neighborhoods for older adults that provide greater opportunities for socialization and formation of social connectedness.

## Supporting information

S1 Checklist Preferred Reporting Items for Systematic reviews and Meta-Analyses extension for Scoping Reviews (PRISMA-ScR) checklist. (DOCX)

## References

[1] Lamblin M, Murawski C, Whittle S, Fornito A. Social connectedness, mental health and the adolescent brain. Neurosci Biobehav Rev. 2017;80:57–68. doi: 10.1016/j.neubiorev.2017.05.010 28506925

[2] Becofsky KM, Shook RP, Sui X, Wilcox S, Lavie CJ, Blair SN. Influence of the source of social support and size of social network on all-cause mortality. Mayo Clin Proc. 2015;90(7):895–902. doi: 10.1016/j.mayocp.2015.04.007 26055526 PMC4492806

[3] Ashida S, Heaney CA. Differential associations of social support and social connectedness with structural features of social networks and the health status of older adults. Journal of Aging and Health. 2008;20(7):872–93. doi: 10.1177/0898264308324626 18815414

[4] Holt-Lunstad J, Robles TF, Sbarra DA. Advancing social connection as a public health priority in the United States. Am Psychol. 2017;72(6):517–530. doi: 10.1037/amp0000103 28880099 PMC5598785

[5] Choi KW, Stein MB, Nishimi KM, Ge T, Coleman JRI, Chen CY, et al. An exposure-wide and mendelian randomization approach to identifying modifiable factors for the prevention of depression. Am J Psychiatry. 2020;177(10):944–954. doi: 10.1176/appi.ajp.2020.19111158 32791893 PMC9361193

[6] Silva M, Loureiro A, Cardoso G. Social determinants of mental health: A review of the evidence. The European Journal of Psychiatry. 2016;(4):259–92.

[7] Bowlby J. *Attachment and loss: Vol. 1. Attachment*. New York: Basic Books; 1982.

[8] Bowlby J. *Attachment and loss*: *Separation*, *anxiety and anger*. New York: Basic Books; 1973.

[9] Cohen S, Wills TA. Stress, social support, and the buffering hypothesis. Psychological Bulletin. 1985;98(2):310–357. 3901065

[10] Thoits PA. Life stress, social support, and psychological vulnerability: Epidemiological considerations. Journal of community psychology. 1982;10(4):341–62. doi: 10.1002/1520-6629(198210)10:4&lt;341::aid-jcop2290100406&gt;3.0.co;2-j 10298894

[11] Hobfoll SE, Freedy J, Lane C, Geller P. Conservation of social resources: Social support resource theory. Journal of Social and Personal Relationships. 1990;7(4):465–78.

[12] Lazarus RS, Folkman S. Stress, appraisal, and coping. Springer Publishing Company; 1984.

[13] Ojagbemi A, Bello T, Gureje O. The roles of depression and social relationships in the onset and course of loneliness amongst Nigerian elders. Int J Geriatr Psychiatry. 2021;36(4):547–557. doi: 10.1002/gps.5451 33091186

[14] Åhlin JK, Rajaleid K, Jansson-Fröjmark M, Westerlund H, Hanson LL. Job demands, control and social support as predictors of trajectories of depressive symptoms. Journal of Affective Disorders. 2018;235:535–43. doi: 10.1016/j.jad.2018.04.067 29689506

[15] Ahmad F, Othman N, Hynie M, Bayoumi AM, Oda A, McKenzie K. Depression-level symptoms among Syrian refugees: findings from a Canadian longitudinal study. J Ment Health. 2021;30(2):246–254. doi: 10.1080/09638237.2020.1765998 32438842

[16] Aroian K, Uddin N, Blbas H. Longitudinal study of stress, social support, and depression in married Arab immigrant women. Health Care for Women International. 2017;38(2):100–17. doi: 10.1080/07399332.2016.1253698 27791495 PMC5607736

[17] Berthelsen M, Pallesen S, Magerøy N, Tyssen R, Bjorvatn B, Moen BE, et al. Effects of psychological and social factors in shiftwork on symptoms of anxiety and depression in nurses: A 1-year follow-up. Journal of Occupational and Environmental Medicine. 2015;57(10):1127–37. doi: 10.1097/JOM.0000000000000532 26461869

[18] Billedo CJ, Kerkhof P, Finkenauer C, Ganzeboom H. Facebook and face-to-face: Examining the short-and long-term reciprocal effects of interactions, perceived social support, and depression among international students. Journal of Computer-Mediated Communication. 2019;24(2):73–89.

[19] Boyden JY, Hill DL, Carroll KW, Morrison WE, Miller VA, Feudtner C. The association of perceived social support with anxiety over time in parents of children with serious illnesses. Journal of Palliative Medicine. 2020;23(4):527–34. doi: 10.1089/jpm.2019.0387 31697175 PMC7364310

[20] Canavan M, Gallo WT, Marshall GL. The moderating effect of social support and social integration on the relationship between involuntary job loss and health. J Appl Gerontol. 2021;40(10):1272–1279. doi: 10.1177/0733464820921082 32536244

[21] Ciarleglio MM, Aslan M, Proctor SP, Concato J, Ko J, Kaiser AP, et al. Associations of stress exposures and social support with long-term mental health outcomes among US Iraq War Veterans. Behavior Therapy. 2018;49(5):653–67. 30146134 10.1016/j.beth.2018.01.002

[22] Crowe L, Butterworth P. The role of financial hardship, mastery and social support in the association between employment status and depression: Results from an Australian longitudinal cohort study. BMJ Open. 2016;6(5):e009834. doi: 10.1136/bmjopen-2015-009834 27235296 PMC4885313

[23] Feldman TR, Carlson CL, Rice LK, Kruse MI, Beevers CG, Telch MJ, Josephs RA. Factors predicting the development of psychopathology among first responders: A prospective, longitudinal study. Psychol Trauma. 2021;13(1):75–83. doi: 10.1037/tra0000957 32940524

[24] Handley TE, Rich J, Lewin TJ, Kelly BJ. The predictors of depression in a longitudinal cohort of community dwelling rural adults in Australia. Social Psychiatry and Psychiatric Epidemiology. 2019;54(2):171–80. doi: 10.1007/s00127-018-1591-1 30155557

[25] Haverfield MC, Ilgen M, Schmidt E, Shelley A, Timko C. Social support networks and symptom severity among patients with co-occurring mental health and substance use disorders. Community Mental Health Journal. 2019;55(5):768–76. doi: 10.1007/s10597-019-00396-7 30863904

[26] Hayslip Jr B, Blumenthal H, Garner A. Social support and grandparent caregiver health: One-year longitudinal findings for grandparents raising their grandchildren. Journals of Gerontology Series B: Psychological Sciences and Social Sciences. 2015;70(5):804–12. 25477430 10.1093/geronb/gbu165

[27] Houtjes W, Deeg D, van de Ven PM, van Meijel B, van Tilburg T, Beekman A. Is the naturalistic course of depression in older people related to received support over time? Results from a longitudinal population‐based study. International Journal of Geriatric Psychiatry. 2017;32(6):657–63. doi: 10.1002/gps.4508 27198491

[28] Misawa J, Kondo K. Social factors relating to depression among older people in Japan: analysis of longitudinal panel data from the AGES project. Aging & Mental Health. 2019;23(10):1423–32. doi: 10.1080/13607863.2018.1496225 30406670

[29] Noteboom A, Beekman AT, Vogelzangs N, Penninx BW. Personality and social support as predictors of first and recurrent episodes of depression. Journal of Affective Disorders. 2016;190:156–61. doi: 10.1016/j.jad.2015.09.020 26519635

[30] Van Den Brink RH, Schutter N, Hanssen DJ, Elzinga BM, Rabeling-Keus IM, Stek ML, et al. Prognostic significance of social network, social support and loneliness for course of major depressive disorder in adulthood and old age. Epidemiology and Psychiatric Sciences. 2018;27(3):266–277. doi: 10.1017/S2045796017000014 28183368 PMC6998855

[31] Porter E, Chambless DL. Social anxiety and social support in romantic relationships. Behavior Therapy. 2017;48(3):335–48. doi: 10.1016/j.beth.2016.12.002 28390497

[32] Scardera S, Perret LC, Ouellet-Morin I, Gariépy G, Juster RP, Boivin M, et al. Association of social support during adolescence with depression, anxiety, and suicidal ideation in young adults. JAMA Network Open. 2020;3(12):e2027491. doi: 10.1001/jamanetworkopen.2020.27491 33275154 PMC7718598

[33] Souto EP, Moreno AB, Chor D, Melo ECP, Barreto SM, Nunes MA, et al. Social capital and depressive episodes: Gender differences in the ELSA-Brasil Cohort. Front Public Health. 2021;9:657700. doi: 10.3389/fpubh.2021.657700 34079785 PMC8165187

[34] Stafford M, Antonucci TC, Zaninotto P. Joint trajectories of spousal social support and depressive symptoms in older age. Journal of Aging and Health. 2019;31(5):760–82. doi: 10.1177/0898264317747077 29254428 PMC6495403

[35] Steine IM, Winje D, Krystal JH, Milde AM, Bjorvatn B, Nordhus IH, et al. Longitudinal relationships between perceived social support and symptom outcomes: Findings from a sample of adult survivors of childhood sexual abuse. Child Abuse & Neglect. 2020;107:104566. doi: 10.1016/j.chiabu.2020.104566 32526550

[36] Whitley DM, Kelley SJ, Lamis DA. Depression, social support, and mental health: A longitudinal mediation analysis in African American custodial grandmothers. The International Journal of Aging and Human Development. 2016;82(2–3):166–87. doi: 10.1177/0091415015626550 26798077

[37] Zhou M, Li F, Wang Y, Chen S, Wang K. Compensatory social networking site use, family support, and depression among college freshman: Three-wave panel study. J Med Internet Res. 2020;22(9):e18458. doi: 10.2196/18458 32795999 PMC7495252

[38] Albuja AF, Lara MA, Navarrete L, Nieto L. Social support and postpartum depression revisited: the traditional female role as moderator among Mexican women. Sex Roles. 2017;77(3–4):209–20. doi: 10.1007/s11199-016-0705-z 28936028 PMC5602525

[39] Asselmann E, Wittchen HU, Erler L, Martini J. Peripartum changes in social support among women with and without anxiety and depressive disorders prior to pregnancy: A prospective-longitudinal study. Archives of Women’s Mental Health. 2016;19(6):943–52. doi: 10.1007/s00737-016-0608-6 26846662

[40] Asselmann E, Kunas SL, Wittchen HU, Martini J. Maternal personality, social support, and changes in depressive, anxiety, and stress symptoms during pregnancy and after delivery: A prospective-longitudinal study. Plos One. 2020;15(8):e0237609. doi: 10.1371/journal.pone.0237609 32833975 PMC7446870

[41] Cankorur VS, Abas M, Berksun O, Stewart R. Social support and the incidence and persistence of depression between antenatal and postnatal examinations in Turkey: A cohort study. BMJ Open. 2015;5(4):e006456. doi: 10.1136/bmjopen-2014-006456 25833665 PMC4390689

[42] Chen HH, Hwang FM, Lin LJ, Han KC, Lin CL, Chien LY. Depression and social support trajectories during 1 year postpartum among marriage-based immigrant mothers in Taiwan. Archives Of Psychiatric Nursing. 2016;30(3):350–5. doi: 10.1016/j.apnu.2015.12.008 27256940

[43] Chen HH, Chien LY. A comparative study of domestic decision-making power and social support as predictors of postpartum depressive and physical symptoms between immigrant and native-born women. PloS One. 2020;15(4):e0231340. doi: 10.1371/journal.pone.0231340 32267897 PMC7141669

[44] Faleschini S, Millar L, Rifas-Shiman SL, Skouteris H, Hivert MF, Oken E. Women’s perceived social support: associations with postpartum weight retention, health behaviors and depressive symptoms. BMC Women’s Health. 2019;19(1):1–8. 31752823 10.1186/s12905-019-0839-6 PMC6873672

[45] Gan Y, Xiong R, Song J, Xiong X, Yu F, Gao W, et al. The effect of perceived social support during early pregnancy on depressive symptoms at 6 weeks postpartum: A prospective study. BMC Psychiatry. 2019;19(1):1–8. 31357958 10.1186/s12888-019-2188-2 PMC6664519

[46] Hagaman A, LeMasters K, Zivich PN, Sikander S, Bates LM, Bhalotra S, et al. Longitudinal effects of perinatal social support on maternal depression: a marginal structural modelling approach. J Epidemiol Community Health. 2021;75(10):936–943. doi: 10.1136/jech-2020-215836 33712512 PMC8434957

[47] Hare MM, Kroll‐Desrosiers A, Deligiannidis KM. Peripartum depression: Does risk versus diagnostic status impact mother–infant bonding and perceived social support? Depression and Anxiety. 2020;38(4):390–99. doi: 10.1002/da.23121 33615587 PMC8026554

[48] Hetherington E, McDonald S, Williamson T, Tough S. Trajectories of social support in pregnancy and early postpartum: Findings from the All Our Families cohort. Social Psychiatry and Psychiatric Epidemiology. 2020;55(2):259–67. doi: 10.1007/s00127-019-01740-8 31256206

[49] Leonard KS, Evans MB, Kjerulff KH, Downs DS. Postpartum perceived stress explains the association between perceived social support and depressive symptoms. Women’s Health Issues. 2020;30(4):231–9. doi: 10.1016/j.whi.2020.05.001 32527464 PMC7347443

[50] Li Y, Long Z, Cao D, Cao F. Social support and depression across the perinatal period: a longitudinal study. Journal of Clinical Nursing. 2017;26(17–18):2776–83. doi: 10.1111/jocn.13817 28334472

[51] Milgrom J, Hirshler Y, Reece J, Holt C, Gemmill AW. Social support—a protective factor for depressed perinatal women? International Journal of Environmental Research and Public Health. 2019;16(8):1426. doi: 10.3390/ijerph16081426 31010090 PMC6518117

[52] Morikawa M, Okada T, Ando M, Aleksic B, Kunimoto S, Nakamura Y, et al. Relationship between social support during pregnancy and postpartum depressive state: A prospective cohort study. Scientific Reports. 2015;5(1):1–9. doi: 10.1038/srep10520 26022720 PMC4448522

[53] Nakamura A, Sutter-Dallay AL, El-Khoury Lesueur F, Thierry X, Gressier F, Melchior M, et al. Informal and formal social support during pregnancy and joint maternal and paternal postnatal depression: Data from the French representative ELFE cohort study. International Journal of Social Psychiatry. 2020;66(5):431–41. doi: 10.1177/0020764020911409 32306806

[54] Ohara M, Okada T, Aleksic B, Morikawa M, Kubota C, Nakamura Y, et al. Social support helps protect against perinatal bonding failure and depression among mothers: A prospective cohort study. Scientific Reports. 2017;7(1):1–8. 28842556 10.1038/s41598-017-08768-3 PMC5572740

[55] Ohara M, Nakatochi M, Okada T, Aleksic B, Nakamura Y, Shiino T, et al. Impact of perceived rearing and social support on bonding failure and depression among mothers: A longitudinal study of pregnant women. Journal of Psychiatric Research. 2018;105:71–7. doi: 10.1016/j.jpsychires.2018.09.001 30205250

[56] Qu J, Weng XL, Gao LL. Anxiety, depression and social support across pregnancy in women with a history of recurrent miscarriage: A prospective study. Int J Nurs Pract. 2021;27(5):e12997. doi: 10.1111/ijn.12997 34342106

[57] Racine N, Plamondon A, Hentges R, Tough S, Madigan S. Dynamic and bidirectional associations between maternal stress, anxiety, and social support: The critical role of partner and family support. Journal of Affective Disorders. 2019;252:19–24. doi: 10.1016/j.jad.2019.03.083 30954841

[58] Racine N, Zumwalt K, McDonald S, Tough S, Madigan S. Perinatal depression: The role of maternal adverse childhood experiences and social support. Journal of Affective Disorders. 2020;263:576–81. doi: 10.1016/j.jad.2019.11.030 31759669

[59] Razurel C, Kaiser B. The role of satisfaction with social support on the psychological health of primiparous mothers in the perinatal period. Women & Health. 2015;55(2):167–86. doi: 10.1080/03630242.2014.979969 25775391

[60] Razurel C, Kaiser B, Antonietti JP, Epiney M, Sellenet C. Relationship between perceived perinatal stress and depressive symptoms, anxiety, and parental self-efficacy in primiparous mothers and the role of social support. Women & Health. 2017;57(2):154–72. 26909523 10.1080/03630242.2016.1157125

[61] Schwab-Reese LM, Schafer EJ, Ashida S. Associations of social support and stress with postpartum maternal mental health symptoms: Main effects, moderation, and mediation. Women & Health. 2017;57(6):723–40. 27104912 10.1080/03630242.2016.1181140 PMC6097234

[62] Senturk V, Abas M, Dewey M, Berksun O, Stewart R. Antenatal depressive symptoms as a predictor of deterioration in perceived social support across the perinatal period: A four-wave cohort study in Turkey. Psychological Medicine. 2017;47(4):766–75. doi: 10.1017/S0033291716002865 27873558 PMC5426317

[63] Tani F, Castagna V. Maternal social support, quality of birth experience, and post-partum depression in primiparous women. The Journal of Maternal-Fetal & Neonatal Medicine. 2017;30(6):689–92. 27123699 10.1080/14767058.2016.1182980

[64] Tsai AC, Tomlinson M, Comulada WS, Rotheram-Borus MJ. Food insufficiency, depression, and the modifying role of social support: Evidence from a population-based, prospective cohort of pregnant women in peri-urban South Africa. Social Science & Medicine. 2016;151:69–77. 26773296 10.1016/j.socscimed.2015.12.042 PMC4766046

[65] Yörük S, Açikgöz A, Türkmen H, Karlidere T. The prevalence of postpartum depression and the correlation of perceived social support and quality of life with postpartum depression: A longitudinal study. P R Health Sci J. 2020;39(4):327–335. 33320462

[66] Yu M, Sampson M, Liu Y, Rubin A. A longitudinal study of the stress-buffering effect of social support on postpartum depression: a structural equation modeling approach. Anxiety Stress Coping. 2021;34(6):751–765. doi: 10.1080/10615806.2021.1921160 33938786

[67] Zheng X, Morrell J, Watts K. Changes in maternal self-efficacy, postnatal depression symptoms and social support among Chinese primiparous women during the initial postpartum period: A longitudinal study. Midwifery. 2018;62:151–60. doi: 10.1016/j.midw.2018.04.005 29684794

[68] Zhong QY, Gelaye B, VanderWeele TJ, Sanchez SE, Williams MA. Causal model of the association of social support with antepartum depression: A marginal structural modeling approach. Am J Epidemiol. 2018;187(9):1871–1879. doi: 10.1093/aje/kwy067 29617921 PMC6118064

[69] Domènech-Abella J, Mundó J, Haro JM, Rubio-Valera M. Anxiety, depression, loneliness and social network in the elderly: Longitudinal associations from The Irish Longitudinal Study on Ageing (TILDA). Journal of Affective Disorders. 2019;246:82–8. doi: 10.1016/j.jad.2018.12.043 30578950

[70] Domènech-Abella J, Mundó J, Switsers L, van Tilburg T, Fernández D, Aznar-Lou I. Social network size, loneliness, physical functioning and depressive symptoms among older adults: Examining reciprocal associations in four waves of the Longitudinal Aging Study Amsterdam (LASA). Int J Geriatr Psychiatry. 2021;36(10):1541–1549. doi: 10.1002/gps.5560 33908639

[71] Evans IE, Llewellyn DJ, Matthews FE, Woods RT, Brayne C, Clare L. Social isolation, cognitive reserve, and cognition in older people with depression and anxiety. Aging & Mental Health. 2019;23(12):1691–700. 30518250 10.1080/13607863.2018.1506742

[72] Förster F, Luppa M, Pabst A, Heser K, Kleineidam L, Fuchs A, et al. The Role of Social Isolation and the Development of Depression. A Comparison of the Widowed and Married Oldest Old in Germany. Int J Environ Res Public Health. 2021;18(13):6986. 10.3390/ijerph18136986 PMC8297151 34210083

[73] Herbolsheimer F, Ungar N, Peter R. Why is social isolation among older adults associated with depressive symptoms? The mediating role of out-of-home physical activity. International Journal of Behavioral Medicine. 2018;25(6):649–57. doi: 10.1007/s12529-018-9752-x 30350258

[74] Holvast F, Burger H, de Waal MM, van Marwijk HW, Comijs HC, Verhaak PF. Loneliness is associated with poor prognosis in late-life depression: Longitudinal analysis of the Netherlands study of depression in older persons. Journal of Affective Disorders. 2015;185:1–7. doi: 10.1016/j.jad.2015.06.036 26142687

[75] Lee SL, Pearce E, Ajnakina O, Johnson S, Lewis G, Mann F, et al. The association between loneliness and depressive symptoms among adults aged 50 years and older: A 12-year population-based cohort study. Lancet Psychiatry. 2021;8(1):48–57. doi: 10.1016/S2215-0366(20)30383-7 33181096 PMC8009277

[76] Martín-María N, Caballero FF, Lara E, Domènech-Abella J, Haro JM, Olaya B, et al. Effects of transient and chronic loneliness on major depression in older adults: A longitudinal study. Int J Geriatr Psychiatry. 2021;36(1):76–85. doi: 10.1002/gps.5397 32791563

[77] Noguchi T, Saito M, Aida J, Cable N, Tsuji T, Koyama S, et al. Association between social isolation and depression onset among older adults: A cross-national longitudinal study in England and Japan. BMJ Open. 2021;11(3):e045834. doi: 10.1136/bmjopen-2020-045834 33737442 PMC7978252

[78] Baek J, Youm Y, Kim HC. Gender differences in the longitudinal association between husbands’ and wives’ depressive symptoms among Korean older adults: the moderating effects of the spousal relationship. Qual Life Res. 2021;30(12):3535–3546. doi: 10.1007/s11136-021-02894-2 34105023

[79] Chang SC, Pan A, Kawachi I, Okereke OI. Risk factors for late-life depression: A prospective cohort study among older women. Preventive Medicine. 2016;91:144–51. doi: 10.1016/j.ypmed.2016.08.014 27514249 PMC5455056

[80] Förster F, Stein J, Löbner M, Pabst A, Angermeyer MC, König HH, et al. Loss experiences in old age and their impact on the social network and depression–results of the Leipzig Longitudinal Study of the Aged (LEILA 75+). Journal of Affective Disorders. 2018;241:94–102. doi: 10.1016/j.jad.2018.07.070 30107351

[81] Reynolds RM, Meng J, Dorrance Hall E. Multilayered social dynamics and depression among older adults: A 10-year cross-lagged analysis. Psychology and Aging. 2020;35(7):948–962. doi: 10.1037/pag0000569 32852977

[82] Santini ZI, Jose PE, Cornwell EY, Koyanagi A, Nielsen L, Hinrichsen C, et al. Social disconnectedness, perceived isolation, and symptoms of depression and anxiety among older Americans (NSHAP): A longitudinal mediation analysis. The Lancet Public Health. 2020;5(1):e62–70. doi: 10.1016/S2468-2667(19)30230-0 31910981

[83] Santini ZI, Jose PE, Koyanagi A, Meilstrup C, Nielsen L, Madsen KR, et al. The moderating role of social network size in the temporal association between formal social participation and mental health: a longitudinal analysis using two consecutive waves of the Survey of Health, Ageing and Retirement in Europe (SHARE). Soc Psychiatry Psychiatr Epidemiol. 2021;56(3):417–428. doi: 10.1007/s00127-020-01961-2 33037448 PMC7904560

[84] Lund C, Brooke-Sumner C, Baingana F, Baron EC, Breuer E, Chandra P, et al. Social determinants of mental disorders and the sustainable development goals: A systematic review of reviews. Lancet Psychiatry. 2018;5(4):357–369. doi: 10.1016/S2215-0366(18)30060-9 29580610

[85] Saeri AK, Cruwys T, Barlow FK, Stronge S, Sibley CG. Social connectedness improves public mental health: Investigating bidirectional relationships in the New Zealand attitudes and values survey. Australian & New Zealand Journal of Psychiatry. 2018;52(4):365–74. 10.1177/0004867417723990 28803484

[86] Zheng S, Masuda T, Matsunaga M, Noguchi Y, Ohtsubo Y, Yamasue H, et al. Cultural differences in social support seeking: The mediating role of empathic concern. PloS One. 2021;16(12):e0262001. doi: 10.1371/journal.pone.0262001 34969056 PMC8718000

[87] Due TD, Sandholdt H, Siersma D, Waldroff FB. How well do general practitioners know their elderly patients’ social relations and feelings of loneliness? BMC Family Pract. 2018;19:34. doi: 10.1186/s12875-018-0721-x 29482509 PMC5828068

[88] Pantell M, Rehkopf D, Jutte D, Syme SL, Balmes J, Adler N. Social isolation: a predictor of mortality comparable to traditional clinical risk factors. Am J Public Health. 2013;103(11):2056–62. doi: 10.2105/AJPH.2013.301261 24028260 PMC3871270

[89] Dykstra PA, van Tilburg TG, de Jong Gierveld J. Changes in older adult loneliness: Results from a seven-year longitudinal study. Res Aging. 2005; 27(6):725–747.

[90] Wrzus C, Wagner J, Hänel M, Neyer FJ. Social network changes and life events across the life span: A meta-analysis. Psychol Bull. 2013; 139(1):53–80. doi: 10.1037/a0028601 22642230

[91] Goossens L, van Roekel E, Verhagen M, Cacioppo JT, Cacioppo S, Maes M, Boomsma DI. The genetics of loneliness: linking evolutionary theory to genome-wide genetics, epigenetics, and social science. Perspect Psychol Sci. 2015;10(2):213–26. doi: 10.1177/1745691614564878 25910391

[92] Tong S, Mullen RA, Hochheimer CJ, Sabo RT, Liaw WR, Nease DE Jr, et al. Geographic Characteristics of Loneliness in Primary Care. Ann Fam Med. 2019;17(2):158–160. doi: 10.1370/afm.2364 30858259 PMC6411395

