# Correction: Social connectedness as a determinant of mental health: A scoping review

**DOI:** 10.1371/journal.pone.0314220

**Published:** 2024-11-15

**Authors:** Priya J. Wickramaratne, Tenzin Yangchen, Lauren Lepow, Braja G. Patra, Benjamin S. Glicksberg, Ardesheer Talati, Prakash Adekkanattu, Euijung Ryu, Joanna M. Biernacka, Alexander Charney, J. John Mann, Jyotishman Pathak, Mark Olfson, Myrna M. Weissman

The fifth author’s name is spelled incorrectly. The correct name is: Benjamin S. Glicksberg. The correct citation is: Wickramaratne PJ, Yangchen T, Lepow L, Patra BG, Glicksberg BS, Talati A, et al. (2022) Social connectedness as a determinant of mental health: A scoping review. PLOS ONE 17(10): e0275004. https://doi.org/10.1371/journal.pone.0275004.
